# AI-powered precision medicine: utilizing genetic risk factor optimization to revolutionize healthcare

**DOI:** 10.1093/nargab/lqaf038

**Published:** 2025-05-05

**Authors:** Sakhaa Alsaedi, Michihiro Ogasawara, Mohammed Alarawi, Xin Gao, Takashi Gojobori

**Affiliations:** Computer Science, Division of Computer, Electrical and Mathematical Sciences and Engineering (CEMSE), King Abdullah University of Science and Technology (KAUST), 23955-6900 Thuwal, Kingdom of Saudi Arabia; Center of Excellence on Smart Health, King Abdullah University of Science and Technology (KAUST), 23955-6900 Thuwal, Kingdom of Saudi Arabia; Center of Excellence for Generative AI, King Abdullah University of Science and Technology (KAUST), 23955-6900 Thuwal, Kingdom of Saudi Arabia; College of Computer Science and Engineering (CCSE), Taibah University, 42353 Madinah, Kingdom of Saudi Arabia; Department of Internal Medicine and Rheumatology, Juntendo University, 113-8431 Tokyo, Japan; Center of Excellence on Smart Health, King Abdullah University of Science and Technology (KAUST), 23955-6900 Thuwal, Kingdom of Saudi Arabia; Center of Excellence for Generative AI, King Abdullah University of Science and Technology (KAUST), 23955-6900 Thuwal, Kingdom of Saudi Arabia; Biological and Environmental Sciences and Engineering, King Abdullah University of Science and Technology (KAUST), 23955-6900 Thuwal, Kingdom of Saudi Arabia; Computer Science, Division of Computer, Electrical and Mathematical Sciences and Engineering (CEMSE), King Abdullah University of Science and Technology (KAUST), 23955-6900 Thuwal, Kingdom of Saudi Arabia; Center of Excellence on Smart Health, King Abdullah University of Science and Technology (KAUST), 23955-6900 Thuwal, Kingdom of Saudi Arabia; Center of Excellence for Generative AI, King Abdullah University of Science and Technology (KAUST), 23955-6900 Thuwal, Kingdom of Saudi Arabia; Center of Excellence on Smart Health, King Abdullah University of Science and Technology (KAUST), 23955-6900 Thuwal, Kingdom of Saudi Arabia; Center of Excellence for Generative AI, King Abdullah University of Science and Technology (KAUST), 23955-6900 Thuwal, Kingdom of Saudi Arabia; Biological and Environmental Sciences and Engineering, King Abdullah University of Science and Technology (KAUST), 23955-6900 Thuwal, Kingdom of Saudi Arabia; Marine Open Innovation Institute (MaOI), 113-8431 Shizuoka, Japan

## Abstract

The convergence of artificial intelligence (AI) and biomedical data is transforming precision medicine by enabling the use of genetic risk factors (GRFs) for customized healthcare services based on individual needs. Although GRFs play an essential role in disease susceptibility, progression, and therapeutic outcomes, a gap exists in exploring their contribution to AI-powered precision medicine. This paper addresses this need by investigating the significance and potential of utilizing GRFs with AI in the medical field. We examine their applications, particularly emphasizing their impact on disease prediction, treatment personalization, and overall healthcare improvement. This review explores the application of AI algorithms to optimize the use of GRFs, aiming to advance precision medicine in disease screening, patient stratification, drug discovery, and understanding disease mechanisms. Through a variety of case studies and examples, we demonstrate the potential of incorporating GRFs facilitated by AI into medical practice, resulting in more precise diagnoses, targeted therapies, and improved patient outcomes. This review underscores the potential of GRFs, empowered by AI, to enhance precision medicine by improving diagnostic accuracy, treatment precision, and individualized healthcare solutions.

## Introduction

**Figure 1.**
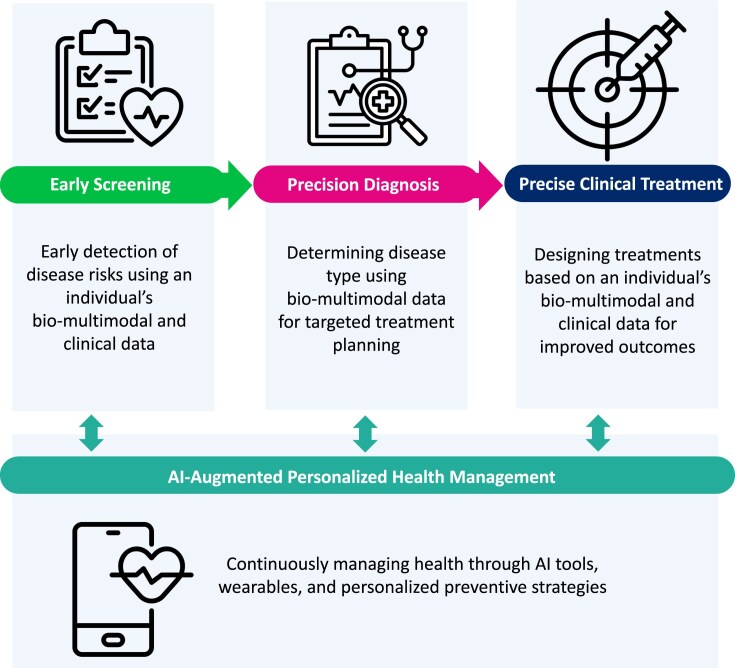
An overview of precision medicine workflow in to customizing healthcare. This figure illustrates the framework of precision medicine, categorized into four key stages: early screening, precision diagnosis, precise clinical treatment, and AI-augmented personalized health management. This approach integrates bio-multimodal and clinical data to enable early detection of disease risks, accurate disease classification, tailored treatment design, and ongoing health management through AI tools and wearable technology.

Artificial Intelligence (AI) is transforming the analysis of omics data, including genomics, transcriptomics, and proteomics, thereby enhancing bioinformatics in precision medicine [1]. Precision medicine is a medical approach that customizes healthcare to the specific needs of individuals [2, 3]. It can be categorized into different stages: early screening, precision diagnosis, precise clinical treatment, and AI-augmented personalized health management, encompassing both healthy individuals and patients, as illustrated in Fig. 1. In this approach, diagnostic testing is frequently employed to select appropriate and optimal medicines based on a patient’s genetic profile or other biochemical or cellular analyses [4, 5]. Currently, most precision medicine interventions involve genetic profiling, which includes the identification of predictive biomarkers and genetic risk factors (GRFs) [6, 7]. This approach can identify patients at risk of certain diseases or severe disease manifestations [5, 8], allowing for preventive interventions that reduce disease complexity and enhance healthcare quality [9]. The fusion of AI and precision medicine is particularly significant in understanding GRFs of diseases [10, 11]. For instance, using AI to integrate different modalities of omics data enhances biomedical knowledge platforms and provides deep epigenetic analysis of disease pathogenicity [10].

Although GRFs represent a significant advancement in healthcare, their current application in precision medicine is limited by several challenges. For example, no clear definition exists for types of GRFs due to their variable expression, complex interactions, ongoing discoveries, and diverse impacts across populations [12]. Moreover, GRFs may not be equally applicable to all diseases, especially complex conditions influenced by multiple genes and environmental factors [13]. Deciphering the complex interplay between GRFs and environmental factors remains difficult, leading to potential misinterpretations and inaccurate risk predictions [14]. Ethical concerns, such as data privacy and security, are paramount in genetic testing [15]. Ensuring informed consent and addressing potential discrimination based on genetic information are crucial. Additionally, the cost and accessibility of genetic testing and personalized treatments can be prohibitive, limiting access for some patients [14].

**Figure 2.**
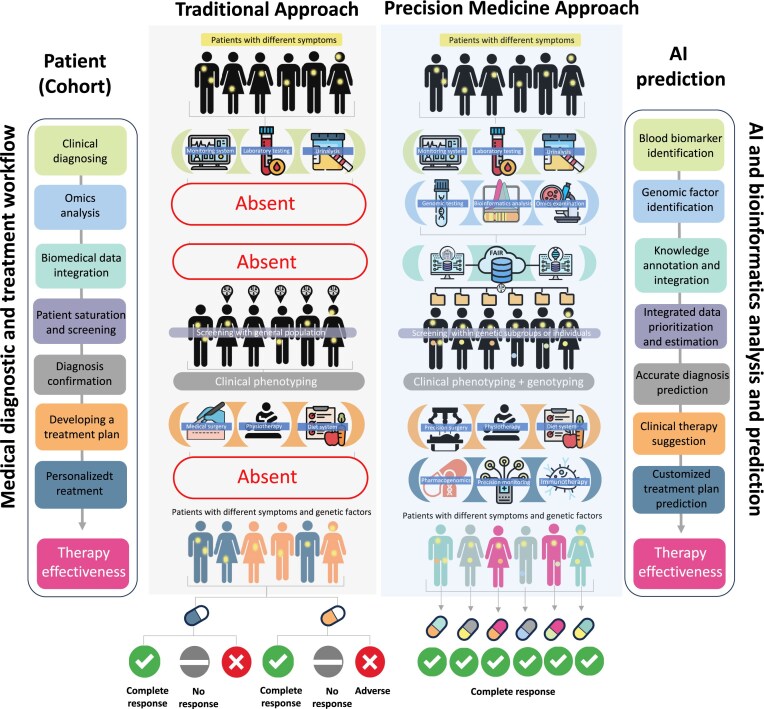
An overview of precision medicine approaches in medical diagnostics and treatment. This figure compares traditional and precision medicine approaches in diagnostics and treatment. The traditional method involves general clinical diagnosing and therapy effectiveness assessment. Precision medicine, however, uses genomic testing, bioinformatics analysis, omics examination, and personalized treatment plans.Dots in the top layer represent symptoms derived from clinical questionnaires, while dots in the middle layer indicate genetic risk factors (GRFs) identified through genetic analysis, which are not included in the traditional approach. Precision medicine integrates patient data with public biomedical resources for accurate diagnosis and personalized care, utilizing AI for better therapy suggestions and improved effectiveness. The diagram illustrates the shift from general to personalized healthcare solutions.

To address such issues, this review demonstrates the applicability of leveraging advanced AI models to tackle challenges related to GRFs and provides potential solutions for solving these issues. It employs AI models to decode and interpret complex genetic information [16], thereby deepening our grasp of individual genetic risk profiles [2]. The rise of AI in precision medicine represents a paradigm shift, enabling healthcare to be customized to each individual’s genome [17]. Through various advanced AI models, case studies, and examples, we illustrate how integrating genetic insights, augmented by AI, into medical practice can lead to more accurate diagnoses, targeted treatments, and improved patient care as shown in Fig. 2. This review emphasizes the role of AI-utilized GRFs in advancing the future of precision medicine and revolutionizing healthcare.

## GRFs in precision medicine

**Figure 3.**
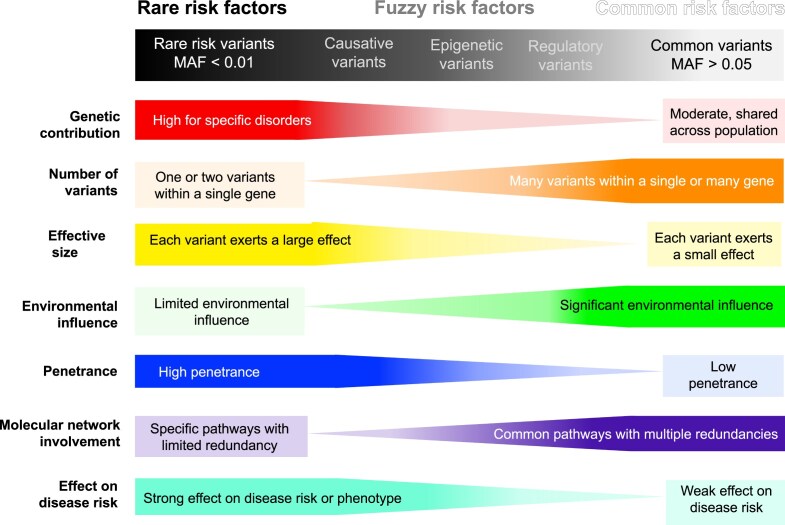
Characteristics of GRFs. This figure illustrates the spectrum of genetic variants contributing to disease risk, with darker colors indicating This figure illustrates the spectrum of genetic variants contributing to disease risk, organized by the strength of their effects. GRFs are categorized into rare variants, which typically have large effects, high penetrance, and minimal environmental influence. These variants often involve specific biological pathways. Common variants have small effects, low penetrance, and are strongly influenced by environmental factors. They usually affect multiple genes and pathways. Fuzzy risk factors, including causative, epigenetic, and regulatory variants, occupy an intermediate space between rare and common variants based on their relative MAF. They demonstrate distinct roles in gene regulation and disease susceptibility.

GRFs are the core of precision medicine, allowing the combination of genetic understanding of disease mechanisms with sophisticated bioinformatic methods for analyzing genetic data [18]. This review considers GRFs at the level of single nucleotide polymorphisms (SNPs) that contribute to disease development or increase disease susceptibility and complexity. Notably, the host genes and proteins associated with these SNPs are also considered risk factors. We categorize GRFs into three categories: rare, common, and fuzzy GRFs. The categorization is based on minor allele frequency (MAF), genetic contribution, functional involvement in molecular networks, number of variants, effective size, environmental influence, penetrance of a disease mutation, and contribution to disease risk and its etiology, as illustrated in Figure 3. This categorization, however, simplifies the complex interactions and continuum of genetic variations, which can result in a nuanced spectrum of risk factors not fully captured by these categories.

### Rare GRFs

Rare genetic factors are specific genetic variants or mutations that occur infrequently in the general population (MAF < 0.01) [19]. These variants often have high penetrance, meaning individuals carrying these rare variants have a high likelihood of developing a particular disease [19, 20]. Rare genetic variants typically exhibit minimal environmental influence but have significant effects on disease risk and phenotype, often directly causing specific diseases or conditions [21, 22]. Despite their high impact, identifying rare variants presents significant challenges due to their low prevalence, necessitating large-scale sequencing studies and often relying on family-based designs [20]. Additionally, the complexities in data interpretation, variant annotation, and computational limitations are considerable hurdles. Although rare variants do not frequently appear in the population compared to common variants, their impact on disease is high and crucial for accurate genetic counseling and precision medicine in affected families [18, 21]. Future research should focus on developing more efficient identification methods and computational tools to better understand these variants.

### Common GRFs

Common genetic factors are specific genetic variants or mutations that occur frequently in the general population, typically with (MAF > 0.05) [21]. These variants have low penetrance and modest effect size, meaning that they individually contribute a small risk to disease development. However, collectively they can significantly influence disease susceptibility and phenotype through polygenic effects [19, 21]. Unlike rare variants, common variants often exhibit substantial environmental interactions, where lifestyle and environmental factors modulate their effects on disease risk [23]. These environmental influences are complex and can significantly vary across different populations and contexts. Common variants are primarily associated with complex, multifactorial diseases such as diabetes, heart disease, and many forms of cancer [24]. These variants are commonly identified through genome-wide association studies (GWAS) [21]. Despite their low individual impact, the cumulative effect of multiple common variants can be substantial, making them essential for understanding the genetic architecture of common diseases and for developing polygenic risk scores (PRS) used in personalized medicine [19, 21]. Identifying and studying common variants provide valuable insights into the genetic basis of complex diseases and inform strategies for prevention, diagnosis, and treatment [23, 24]. Their high frequency in the population makes them useful markers for identifying population-wide risk factors and understanding the etiology of diseases [22]. Comprehensive integration of environmental data with genetic data is crucial for a deeper understanding of gene-environment interactions.

### Fuzzy GRFs

Fuzzy GRFs represent a category of genetic variants with MAFs between rare and common variants (0.01–0.05). These variants often have intermediate frequencies and variable effects on disease risk or phenotype [25]. Fuzzy genetic factors include causative, regulatory, and epigenetic variants, which play distinct yet interconnected roles in genetic and phenotypic diversity [26]. The identification and study of these fuzzy variants require advanced techniques beyond genome sequencing and GWAS, such as chromatin immunoprecipitation sequencing, RNA sequencing (RNA-seq), and bisulfite sequencing, to capture the dynamic nature of gene regulation and epigenetic modifications [26]. Recognizing and studying fuzzy genetic factors is essential for understanding the nuanced genetic architecture of multifactorial diseases [26, 26]. The intricate interplay between these variants and their ability to modulate gene expression and phenotype highlights their importance in precision medicine and personalized treatment approaches [3, 11]. However, the probabilistic nature of their effects and the challenges in studying their interactions with environmental factors and other genetic variants should be emphasized.

#### Causative variants

Causative variants are genetic alterations that directly contribute to disease or specific phenotypes by altering gene function or expression [27]. These variants are often responsible for monogenic or Mendelian disorders, where a single genetic mutation leads to a specific illness [28]. Notably, some causative variants can also be common; for instance, certain variants in the Hemoglobin Subunit Beta (HBB) gene confer resistance to malaria while causing sickle cell anemia in homozygous individuals [29]. Understanding the full spectrum of causative variants, including their varying frequencies and impacts, is essential for comprehensive genetic counseling and precision medicine strategies.

#### Regulatory variants

Regulatory variants influence gene expression by modifying regulatory elements such as promoters, enhancers, and silencers, thereby affecting the timing, location, and level of gene activity [26, 30]. These variants play a critical role in the fine-tuned regulation of gene expression and shaping complex trait architecture. For example, variants in the enhancer regions of the Fat mass and obesity-associated (FTO) gene that are associated with fat mass and obesity have been linked to obesity by altering the expression of genes involved in fat metabolism [31]. The dynamic and context-dependent nature of regulatory variants, influenced by both genetic and environmental factors, underscores the need for advanced analytical methods to study their effects comprehensively.

#### Epigenetic variants

Epigenetic variants change gene function without altering the DNA sequence [32], modifying the epigenome through mechanisms such as DNA methylation [33], histone modification [34], and non-coding RNA interactions [35]. The interaction of these variants with environmental factors often makes their effects on disease risk and phenotype complex and variable [32]. As such, epigenetic variants provide an important link between genetic predisposition and environmental influences. The study of these variants requires an integrative approach, combining genetic, epigenetic, and environmental data to understand their multifaceted roles in health and disease [26].

## AI methods for utilizing GRFs in precision medicine

**Figure 4.**
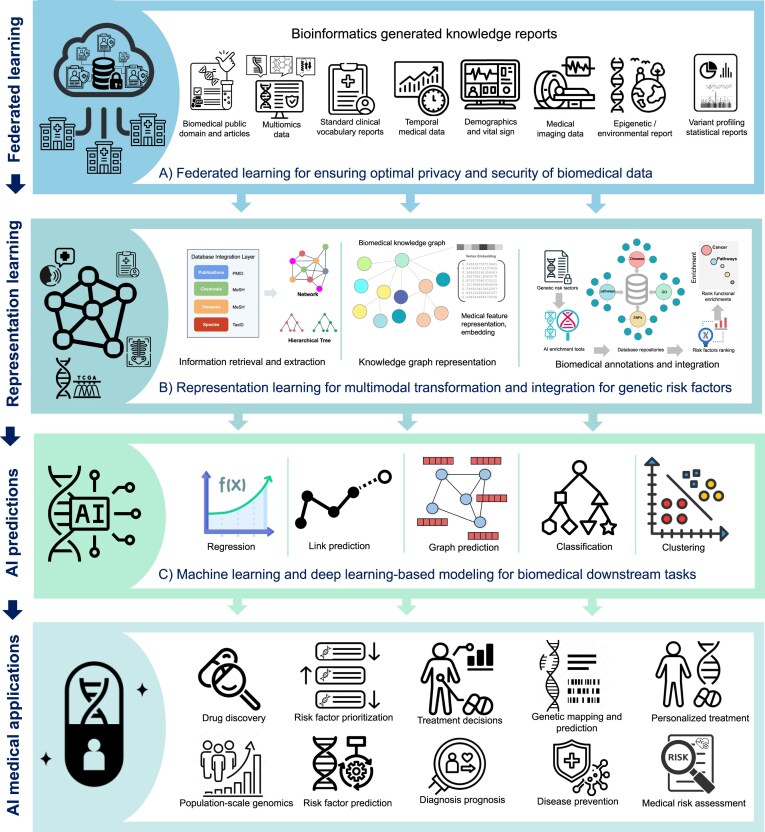
AI-based biomedical applications for utilizing GRFs in medical care. This figure illustrates the utilization of medical data resources with advanced AI models to provide accurate applications for different medical tasks. It encompasses: (**A**) Federated learning for ensuring optimal privacy and security of biomedical data, (**B**) Representation learning for multimodal transformation and integration of GRFs, and (**C**) ML and DL modeling for biomedical downstream tasks. The figure highlights the use of AI to enhance treatment decisions, medical risk assessment, drug discovery, and personalized treatment by leveraging genetic mapping, risk factor prediction, and other biomedical data.

AI enhances experimental [50], computational [25, 51, 52], and genetic techniques [53] to uncover the genetic basis of diseases, enabling more effective therapies [35, 54]. These AI models utilize biomedical big data from various modalities such as text, images, tabular data, and graphs to train their models for performing different downstream tasks. Recent advancements in AI methods, including statistical models [36], federated learning [37, 38], machine leaning (ML) [39–41], and deep learning (DL) methods [42, 43] such as generative models [44], representation models [45, 46], and language models [47, 48] have significantly expanded the potential of personalized medicine [49]. Fig. 4 illustrates how AI integrates data from multiple biomedical modalities to identify risk factors and apply them in precision medicine.

### Data privacy and cost-effectiveness in biomedical data

The biomedical data resources are placed in the top layer of Fig. 4, highlighting their critical role in advancing AI applications. High-quality datasets such as the Protein Data Bank (PDB), UK Biobank, and TCGA have enabled significant advancements in protein prediction, genomics, and precision medicine by providing diverse, large-scale data for AI training. Sharing these datasets fosters collaboration, improves model performance, and accelerates diagnostic and therapeutic progress. However, sharing sensitive biomedical and genetic data raises significant privacy concerns. Patients’ medical and genetic information must be protected against misuse, while still enabling data sharing for research [38, 50].

To address these requirements, federated learning algorithms are developed to encrypt sensitive data [38, 50]. For instance, PPML-Omics employs decentralized differential privacy techniques to securely analyze multi-omics data while preserving patient confidentiality [37, 51]. Following Findable, Accessible, Interoperable, and Reusable principles (FAIR) [52], federated learning workflows provide secure channels that encourage patients to share their genetic data [37, 51]. EUGene, a FAIR toolkit, simplifies DL analysis of genomic sequences, aiding in tasks such as gene function prediction and disease variant analysis [53]. This method is cost-effective for enhancing research using public encrypted data for better risk identification [54, 55], early disease detection [56], and customized treatments in complex diseases [57]. Federated learning allows hospitals and clinics to securely share patient biomedical and genetic records, enhancing healthcare outcomes by integrating genetic factors, clinical records, and lifestyle information into precision medicine [58]. Tools such as Decagon and PathGNN further enhance drug discovery and disease modeling using such datasets [59]. Moreover, AI enhances data privacy while advancing biomedical research by providing secure solutions for working with sensitive datasets. Platforms such as Watson for Genomics and initiatives such as the Human Cell Atlas and GenBank offer high-quality, curated datasets for training AI models, driving innovation across diverse biomedical applications. Sharing private datasets under FAIR principles is essential for enabling precision medicine, fostering collaboration, and advancing diagnostics and treatments.

### Biomedical data integration and knowledge representation

Integrating and structuring biomedical data is crucial for enriching features, enhancing AI model predictions, ensuring reproducibility, and deriving insights. AI methods utilize advanced data mining and retrieval techniques to extract significant information from platforms such as PubMed and MedPlus. For instance, text mining and retrieval methods, such as SNPMap and LitVar, play a key role in automating variant-related literature searches by visualize and retrieve disease-linked genetic factors. These tools link genetic variants to diseases and chemicals by leveraging resources such as PubMed and PubTator. By utilizing advanced text mining techniques, they provide comprehensive, up-to-date, and efficient semantic searches, significantly enhancing the accessibility and usability of biomedical knowledge for research and clinical applications.

In addition, integrating multi-omics data with risk factors facilitates the identification of risky molecular pathways and highlights potential drug targets for complex diseases [60]. This approach navigates the intricate molecular pathways of genes that contribute to the complexity of disease. DeepHisCoM [61] analyzes multi-omics data, GWAS data, and ontologies to predict pathway effects in complex diseases [1]. ExPecto [62] leverages DL to predict the impact of genetic variants on gene expression and disease risk. AI unlocks the power of complex biomedical data by integrating multi-omics information with annotated resources such as the Open Targets Platform [63] and CasualDB and more annotated databases [64].

Although maintaining and integrating data enriches AI models, challenges such as sparsity and inconsistency remain. Transforming raw data into structured formats helps uncover the relationships between variants, diseases, and molecular networks, addressing the complexities of multifactorial diseases. The third stage in Fig. 4, knowledge representation learning, structures the data to enhance AI-driven insights [65]. For example, disease knowledge graphs integrate diverse datasets and systematically reveal the interplay of genetic, molecular, and environmental factors that influence disease progression [66]. Representation learning with AI extracts patterns from raw data and organizes them into knowledge graphs, linking entities such as genes, proteins, drugs, and phenotypes [65]. These graphs provide a unified framework for exploring molecular interactions, causal disease mechanisms, and treatment pathways [67].

Knowledge representation learning is essential for advancing biomedical research, as it structures complex datasets into actionable frameworks [67]. Knowledge graphs, in particular, enable the systematic representation of interconnected entities, such as genes, proteins, and diseases, allowing researchers to uncover hidden relationships and patterns [66]. BSuch biomedical knowledge representations in personalized medicine enhance the processing of complex data patterns, improvising the accuracy of risk prediction by handling complex data patterns [68]. These graphs integrate diverse datasets, including genomic variants, molecular networks, and disease phenotypes, into unified systems, offering a comprehensive view of multifactorial diseases. By leveraging advanced models such as Graph Convolutional Networks (GCNs), Graph Attention Networks (GATs), and embedding techniques such as Node2Vec, knowledge representation frameworks support predictive analysis, facilitating tasks such as risk assessment, biomarker discovery, and pathway exploration.

AI-driven knowledge graphs excel in integrating multi-modal datasets, enabling researchers to study diseases from a systemic perspective. Diseases are influenced by multiple genetic, environmental, and molecular factors, making traditional methods insufficient for fully capturing their complexity. Knowledge graphs, such as STRING, DisGeNET, and Open Targets, reveal intricate relationships between genetic variants, molecular networks, and disease mechanisms. For example, STRING maps protein-protein interactions, while DisGeNET connects genetic variants with disease phenotypes, aiding in the identification of critical molecular pathways. This capability supports cross-disciplinary research by bridging data modalities such as transcriptomics and proteomics, uncovering critical insights into disease mechanisms and enabling the discovery of therapeutic targets.

In functional genomics and disease research, AI-powered knowledge graphs enhance gene interaction analysis by modeling regulatory networks and their impact on gene expression. By incorporating tissue-specific data from resources such as ENCODE and GTEx, these graphs reveal how non-coding variants influence gene regulation [65]. For instance, GCNs applied to STRING data can predict novel gene-disease associations or highlight key regulatory elements in transcriptional pathways.

Applications such as PASNet use multi-omics data for prognosis prediction, while tools such as Decagon synthesize multi-modal information to identify drug targets and repurpose treatments [69]. By systematically capturing complex relationships, knowledge graphs drive biomarker discovery, prioritize therapeutic targets, and facilitate personalized medicine [46]. For example, SNPMap clarifies disease-linked genetic factors by extracting and visualizing SNP data from various sources. MecCog is a graphical knowledge framework that integrates genetic variants and disease mechanisms for causative biomarker and treatment discovery [46]. PathGNN captures pathway features as biomedical knowledge graphs to predict cancer survival [59], while PASNet uses high-throughput data for prognosis prediction in somatic variants [69]. Decagon integrates genetic risks, causative factors, and multi-omics data for lower-dimensional representations, aiding medical decisions [70]. DL platforms, such as Gene Ontology, Pathology Workbench, and Watson for Genomics translate complex genetic risks, empowering healthcare professionals and patients [66]. AI streamlines the abundance of information for easy access and comprehension within biomedical knowledge representation. Their ability to translate diverse datasets into actionable insights demonstrates their potential in advancing precision medicine and accelerating breakthroughs in disease understanding [66].

### Comparative overview of ai-powered computational downstream tasks in biomedical applications

Stages 3 and 4 of Fig. 4 outline the main computational tasks that process biomedical data into insights for GRFs analysis and prediction. The main tasks include regression, link prediction, graph representation, clustering, and classification, each defined by its computational method and application.

Regression identifies continuous relationships between input features and outputs, such as predicting genetic risk scores or disease progression. Tools such as PRSice, DeepWAS, and PolyPred effectively calculate PRSs, leveraging statistical models and DL for high-dimensional genomic data [19, 71]. Link prediction evaluates associations in structured datasets, such as gene-disease or protein-protein interactions. Methods such as SNPMap and GRAM utilize graph-based algorithms to infer meaningful relationships, while MiRLog and dbmiR focus on prioritizing interactions involving microRNA variants [72]. Graph representation encodes complex biomedical relationships into structured networks. Approaches such as GRAM, MecCog, and SNPMap integrate data into graphs for tasks like pathway analysis and variant visualization [72]. Tools such as VariantSpark analyze large-scale multi-omics data to reveal disease-associated variants through graph-based embeddings [73]. Clustering uncovers inherent patterns by grouping data based on similarity metrics or latent spaces. Tools such as DeepHisCoM and MUSSEL reveal subpopulations or regulatory elements linked to diseases [74]. Methods such as RegBase apply clustering to annotate and predict non-coding regulatory variants with high precision. Classification assigns entities to predefined categories, such as pathogenic or benign variants. Tools such as CADD, EVE, and Xrare excel in variant classification, providing high accuracy in identifying pathogenic or rare disease-associated variants [44]. Similarly, tools such as DeepSEA and RegVar prioritize functional impact predictions for coding and non-coding genomic regions [36, 62].

In Stage 4 of Fig. 4, several applications are enhanced by leveraging computational tasks in GRF analysis, including risk assessment, early prevention, disease diagnosis, treatment prediction, and drug discovery. For instance, predicting the functional effects of genetic variants is essential for understanding gene regulation and disease mechanisms [75–78]. Classical in silico prediction methods, such as FATHMM, utilize hidden Markov models to assess the functional impacts and pathogenicity of protein missense variants [79]. Similarly, PolyPhen-2 employs sequence and structure-based features combined with a naive Bayes classifier to predict the effects of protein-coding genetic variants. Moreover, ASpediaFI identifies differential alternative splicing events and co-regulated genes to predict the functional effects of risk variants using an interaction network and random walk algorithm. DL-based methods such as DeepSEA and DeepCAGE utilize CNNs) to predict chromatin features, transcription factor binding, and variant effects from GWAS statistical reports, providing insights into gene regulation and potential disease mechanisms [80]. ExPecto use CNNs to predict tissue-specific transcriptional effects of mutations. RegVar predict tissue-specific regulatory impacts of non-coding variants [36, 62].

When decoding functional impact, AI algorithms integrate various ML and statistical methods to identify the pathogenicity of variants. OncoBase [81] has decoded regulatory somatic mutations in cancer, and X-CNV [82], StrVCTVR [83], and ClassifyCNV [84] have identified the copy number of variants based on. Additionally, parSMURF [85] tackles imbalanced data in pathogenic variant detection using ML with oversampling and undersampling techniques. Public genomics resources combined with the integration of multiple annotated datasets provide another approach. ML models such as FAVOR [86], Funseq2 [87], and Deep post-GWAS [80] leverage this strategy, integrating various omics data to pinpoint risk genes and variants associated with complex diseases [88]. iMEGES, a DL model [89] analyzes whole-genome sequencing data to pinpoint potential risk genes for mental disorders, aiding in both population studies and personalized medicine.

For coding variants, ESM1b uses a protein language model to predict the effects of coding variants. It also annotates variants as damaging only in specific protein isoforms and generalizes to complex coding variants [48]. These tools advance precision medicine by enhancing the understanding of gene regulation in different tissues.

Moreover, prioritizing causative mutations and pathogenic variants is vital for understanding disease mechanisms [66]. Fine mapping methods, such as FINEMAP [90] and PICS2 [91], optimize resource allocation by identifying causal factors from GWAS data [92, 93]. AI-driven tools, such as MUFFIN [94], integrate data for functional annotation of genetic variants, supporting the identification of disease-linked mutation for pinpointing causative mutations in diseases [66, 88] and predicting variant pathogenicity and causative effects [43, 93].

In the context of risk assessment and early prevention, AI models enhance GWAS by automating the analysis of vast genomic datasets. Methods such as EigenGWAS employ eigenvector decomposition to identify genetic associations [95]. DeepWAS uses CNNs for accurate SNP identification [71]. These advancements significantly contribute to precision medicine by supporting personalized disease risk prediction and prevention. Such AI-driven methods enhance the prediction of disease risk, addressing the complexity of genetic interactions and environmental influences in precision medicine.

The computational tasks in GRF analysis cater to different aspects of genetic research, with tools excelling in specific domains. Regression tools like PRSice and DeepWAS are effective for polygenic risk scoring, leveraging both statistical models and DL for high-dimensional data. Link prediction tools such as GRAM and SNPMap excel in uncovering associations, while graph representation methods like VariantSpark and MecCog provide structured insights into complex relationships. Classification tools, including CADD and Xrare, are highly accurate for identifying pathogenic variants, and clustering approaches like DeepHisCoM reveal hidden patterns in regulatory elements. While traditional tools like FATHMM and PolyPhen-2 are lightweight and interpretable, DL models like DeepSEA and ExPecto offer unparalleled accuracy for functional impact prediction but at a higher computational cost. Overall, the choice of tools depends on the trade-off between computational demand and the specificity of the task, with advanced AI models offering deeper insights for complex analyses.

## Comparative evaluation of AI models for risk prediction and estimation

To streamline understanding, we focus on four core downstream tasks that form a comprehensive workflow for GRFs analysis: risk variant annotation, prioritization, estimation, and interpretation. A comparative evaluation of 15 methods out of 20 across these tasks, including non-AI approaches such as statistical models [19, 71] and AI approaches such as such as ML and DL models [39, 41–43], highlights their strengths and limitations based on performance, complexity, robustness, and scalability. Moreover, we evaluated these methods and ranked them (1–5) based on five significant criteria: experimental validation, computational resource optimization, model performance and novelty, flexibility and integration with other tools, and scalability to other biomedical tasks. For example, this includes applying analyses of different DNA regions to other datasets or applications. Table 2 provides a comprehensive analysis and evaluation of these 15 methods.

**Table 1.** List of AI methods for optimizing GRFs in precision medicine

Method	Methodology	Input	Output	Biomedical application	Clinical validation	Implementation	PMID
DeepSEA	Convolution neural network	Genomic sequences	Predicted chromatin features, transcription factor binding, and variant effects	Identifying functional and chromatin effects of noncoding variants	Yes (Experimental validation)	Platform	26301843
DeepWAS	Convolutional neural networks	Genotype and regulatory info (chromatin features, cell types)	SNPs associated with phenotypes	Fine-mapping GWAS associations and identifying disease mechanisms	No (Only computational validation)	Github	32012148
VariantSpark	Random forest	Genotype data, complex phenotype data	Variants associated with complex phenotypes	Identifying genomic variants associated with complex phenotypes	No (Only computational validation)	Github	32761098
Xrare	Gradient boosting decision tree	Genetic sequences, clinical phenotypes	Causative variant prediction	Prioritizing rare disease-associated variants	Yes (Real clinical datasets)	N.A	30675030
WEVar	Weighted ensemble of multiple scoring methods	Non-coding regulatory variants (SNVs)	Functional and pathogenic predictions	Predicting regulatory impact of non-coding variants	Yes (Multiple risk loci validation)	Github	34021560
MecCog	Graphical notations	Genetic variant data, biomedical literature	Integrated representations of disease mechanisms, identification of biomarkers, therapeutic intervention sites	Understanding genetic disease mechanisms and identifying potential biomarkers and therapeutic targets	Yes (Real clinical data and expert feedback)	Web server	34117883
DeepSNP	Neural network with attention-based localization	Genome-wide SNP array data	Breakpoints detection within large genomic windows	Identifying disease-associated CNVs in cancer and other diseases	No (Only computational validation)	Github	30585743
RegVar	Deep neural network	Non-coding genomic variants, gene expression data	Tissue-specific regulatory impact predictions, prioritized variants	Prioritizing non-coding regulatory variants in various tissues	No (Only computational validation)	Web server	34973416
SNPMap	Automated pipeline, data integration	Text-mined SNP-related info, GWAS Catalog	Visual interpretation of SNPs, prioritized variants	Interpreting and visualizing single-nucleotide polymorphisms (SNPs) in precision medicine	No (Only computational validation)	N.A	36061173
EUGene	Convolutional neural networks	Genomic sequences	Predictive analyses of regulatory sequences, identification of sequence features	Predicting regulatory activity and understanding cis-regulatory code	No (Only computational validation)	Github	38177592
GREEN-DB	Various ML and statistical methods	Whole-genome sequencing data	Annotated regulatory variants, prioritized variants	Prioritizing non-coding regulatory variants	Yes (Experimental validation)	Github	35234913
PolyPred	Combination of PolyFun-pred and BOLT-LMM	Genotyped and imputed data	PRSs, cross-population prediction accuracy	Genetic risk prediction and addressing linkage disequilibrium differences	Yes (Experimental validation)	Github	35393596
CADD	SVM, ensemble methods	Genetic variants (SNVs, indels)	Scaled and raw CADD scores indicating variant deleteriousness	Prioritizing causal variants and identifying deleterious variants for Mendelian disorders	Yes (Experimental validation)	Github	30371827
PRS-ML	Combining SNP selection and XGBoost	Genetic variants (SNPs), PRS)	PRSs	Predicting genetic risk for complex diseases	Yes (Experimental validation)	Github	35995843
Geneformer	Transformer models	Single cell transcriptomes	Context-specific predictions	Discovering key network regulators, candidate therapeutic targets	Yes (Experimental validation)	N.A	37258680
GRAM	LASSO, random forest	Non-coding variants, TF binding scores, gene expression data	Expression-modulating effects of non-coding variants	Predicting molecular effects of non-coding variants in a cell-type specific manner	Yes (Experimental validation)	Github	31469829
DSNetwork	Integration of multiple prediction scores	Genetic variants	Prioritized variants	Prioritizing and visualizing variant predictions	Yes (Experimental validation)	N.A	32010198
MiRLog and dbmiR	Logistic regression	microRNA SNVs	Deleteriousness scores, functional annotation	Prioritizing and annotating miRNA sequence variants	Yes (Experimental validation)	Github	35583122
RegBase	Gradient tree boosting	Non-coding regulatory variants (SNVs)	Pathogenic and cancer driver predictions	Functional annotation, pathogenicity prediction	Yes (Experimental validation)	Github	31511901
IMPUTE2	Hidden Markov model, Markov chain Monte Carlo	Genomic variants	Imputed genotypes for untyped SNPs	Imputation in GWAS	Yes (Real clinical data and expert feedback)	Web server	34117883

**Table 2.** Comparison of AI tools for genomic analysis, including computational resources, performance metrics, and rankings

Tool	AI model	Downstream task	Purpose	Strengths	Limitations	Computational resources	Performance	Rank (1–5)
DeepSEA	DL: CNN	Functional annotation	Annotate the functional impact of variants	Integrates regulatory genomic data	Limited tissue specificity	Moderate ( 16 GB RAM)	>90% AUROC accuracy in chromatin feature prediction	5
LINSIGHT	ML: Linear Model	Functional annotation	Score non-coding variant impacts	Integrates evolutionary and functional data	Limited contextual specificity	Low (standard laptop resources sufficient)	91% AUC for non-coding deleteriousness prediction	4
MiRLog	ML: Logistic Regression	Functional annotation	Annotate miRNA sequence variants	Effective for miRNA-related studies	Limited to miRNA datasets	Low (standard laptop resources sufficient)	>85% average AUC for functional annotation in miRNA regions	4
DeepVariant	DL: CNN	Functional annotation	Detect and annotate genetic variants	High accuracy; widely validated	Computationally intensive	High (requires > 32 GB RAM, GPU recommended)	>99% SNP accuracy	3
DeepSNP	DL: Attention-based Neural Network	Functional annotation	Detect structural variants	High precision for structural variations	Lacks experimental validation	Moderate ( 16 GB RAM)	90% macro F1-score in structural variant detection	3
RegVar	DL: DNN	Genetic risk prioritization	Prioritize tissue-specific non-coding variants	Strong tissue-specific prioritization	High-quality tissue data required	High (requires >32 GB RAM, GPU recommended)	96% AUC in predicting regulatory variants; >88% AUROC in distinguishing pathogenic variants	4
FINEMAP	SL: Bayesian Variable Selection	Genetic risk prioritization	Identify causal genetic variants	Handles large datasets efficiently	Assumes linear genetic architecture	Moderate ( 16 GB RAM)	99% accuracy in fine-mapping and causal variant prediction	4
DeepWAS	DL: CNN	Genetic risk prioritization	Fine-map GWAS associations	Strong multivariate associations	High computational costs	High (requires >32 GB RAM, GPU recommended)	>90% accuracy in GWAS signal recovery	4
CADD	SL: Ensemble Learning	Genetic risk prioritization	Identify deleterious variants	Widely validated; integrates multiple annotations	Limited to predefined annotations	Moderate ( 16 GB RAM)	98% accuracy for deleteriousness and pathogenic variant prediction	5
GREEN-DB	Various ML Models	Genetic risk prioritization	Annotate regulatory variants using multi-omics	Robust annotation framework	Requires comprehensive multi-omics data	Moderate ( 16 GB RAM)	90% annotation reliability	4
WEVar	Statistical learning	Risk estimation	Predict regulatory impact of variants	Incorporates multiple scoring methods	Newer tool with limited benchmarks	Low (standard laptop resources sufficient)	>91% AUROC in non-coding functional prediction	4
GRAM	ML: Random Forest	Risk estimation	Predict molecular effects of non-coding variants	High specificity for cell-type analysis	Computational costs are significant	Moderate ( 16 GB RAM)	94% AUC with high specificity in cell-type predictions	4
Xrare	ML: Gradient Boosting Decision Tree	Risk estimation	Prioritize rare disease-associated variants	Effective for clinical applications	Limited to rare diseases	Low (standard laptop resources sufficient)	95% average accuracy for rare variants	4
EVE	DL: Generative Model	Risk estimation	Predict pathogenicity of rare variants	Effective for rare variants	Limited to protein-coding regions	Moderate ( 16 GB RAM)	91% average AUC for rare pathogenic variants	4
PolyPred	SL: Linear Mixed Model + Polygenic Models	Risk estimation	Predict genetic risk across populations	High cross-population accuracy	Requires imputed datasets	Moderate ( 16 GB RAM)	>92% average AUC for PRS across populations	4
PRSice	Statistical linear models	Risk estimation	Calculate PRSs	Flexible; handles large datasets	Computationally intensive for large-scale cohorts	Moderate ( 16 GB RAM)	High PRS prediction accuracy	2
MecCog	Graphical model	Visualization and interpretation	Understand disease mechanisms	Integrates biomedical literature and real-world data	Requires expert curation	Low (standard laptop resources sufficient)	90% biomarker identification accuracy	4
DeepHisCoM	DL: CNN	Visualization and interpretation	Analyze pathways using multi-omics data	Integrates multi-omics data	Requires extensive training data	High (requires >32 GB RAM, GPU recommended)	94% pathway analysis precision	4
SNPMap	SL: Automated Pipeline	Visualization and interpretation	Visualize and interpret SNP effects	User-friendly; integrates data from multiple sources	Limited to pre-existing annotations	Low (standard laptop resources sufficient)	85% accuracy in SNP interpretation	4
VariantSpark	ML: Random Forest Ensemble	Visualization and interpretation	Identify complex trait associations	Handles complex datasets and SNP interactions	Less interpretable; requires significant resources	High (requires >32 GB RAM)	85% prediction accuracy for complex traits	2

### Task 1: GRF annotation and identification

Non-coding regions influence gene expression and surpass coding regions in disease risk, highlighting their importance in identifying risk variants. We compare methods targeting and annotating regulatory elements.

DeepSEA uses CNNs to predict chromatin accessibility, histone marks, and transcription factor binding for non-coding variants. It achieves a high AUROC of ∼0.9 and integrates cell-type-specific features but requires significant computational resources for large-scale analyses [62]. This makes it ideal for functional annotation of regulatory variants and fine-mapping studies. LINSIGHT combines evolutionary conservation with functional genomic data to prioritize non-coding variants based on their fitness impact. With a high area under curve (AUC) value of ∼0.91 and low computational requirements, it is efficient and scalable for population-based studies. However, it lacks tissue-specific resolution and struggles in rapidly evolving genomic regions, limiting its versatility [96]. MiRLog specializes in miRNA-specific analysis, prioritizing variants affecting miRNA binding and gene expression. It integrates multiple deleteriousness scores and achieves >85% AUC for miRNA variant prioritization. Its low computational requirements make it accessible, but its generalizability is constrained by small training datasets, limiting its use to miRNA-associated studies [72].

Each tool addresses different aspects of GRFs analysis: DeepSEA is the most versatile for regulatory and coding variant analyses when resources allow, LINSIGHT is ideal for non-coding fitness-based prioritization, MiRLog excels in miRNA studies. The choice of tool depends on the research focus, computational resources, and dataset scope. DeepSEA is the most robust overall tool, and its limitations can be mitigated by integrating missing features from LINSIGHT to improve fitness-based filtering. Downstream analysis can be further refined by using LINSIGHT tool for high-throughput filtering and MiRLog for miRNA-specific variant filtering. Computational resource demands can be reduced by pre-filtering variants with LINSIGHT before applying DeepSEA for detailed analyses.

### Task 2: GRF prioritization

FINEMAP is designed for causal SNP identification in GWAS studies using Bayesian probabilistic models [90]. It achieves 99% accuracy in predicting causal variants, making it one of the most reliable tools for fine-mapping GWAS associations. Requiring moderate computational resources, FINEMAP efficiently handles large GWAS datasets, integrates LD information, and explores causal configurations effectively. However, its accuracy depends heavily on the quality of LD matrices and GWAS summary statistics. Additionally, FINEMAP cannot directly integrate functional annotations, such as regulatory or epigenetic data, limiting its scope. RegVar identifies and prioritizes tissue-specific regulatory variants linked to non-coding pathogenic variants [36, 62]. It delivers 96% AUC in regulatory variant prediction, excelling in combining genomic, epigenetic, and evolutionary data for robust tissue-specific impact predictions. Supporting both common and rare variants, RegVar is particularly strong for non-coding variant analysis. However, it is computationally intensive and is heavily dependent on high-quality tissue-specific data. Furthermore, it struggles to predict impacts in neighboring regions, which can limit its performance in broader analyses. DeepWAS integrates functional annotations with GWAS results to prioritize SNPs and link them to phenotypes or cell-type-specific mechanisms. Achieving 90% GWAS signal recovery accuracy, it effectively bridges GWAS and functional data, reducing the multiple testing burden associated with traditional GWAS studies. However, DeepWAS is computationally demanding and reliant on the completeness of functional data, such as ENCODE. Its applicability can be limited in underpowered GWAS datasets, which hinders its use in studies with smaller sample sizes or incomplete annotations. CADD prioritizes deleterious and causal variants for Mendelian and complex traits using ensemble learning. With 98% accuracy for pathogenic variant prediction, CADD integrates over 60 genomic features, combining functional and evolutionary annotations for comprehensive prioritization. It supports both coding and non-coding variant analysis, providing reliable scoring across the genome [47, 48]. Unlike RegVar and DeepWAS, CADD requires moderate computational resources and is scalable for genome-wide analyses. However, it cannot predict tissue-specific impacts or long-range regulatory effects, and its intermediate scores lack interpretability.

When comparing these methods, FINEMAP stands out for causal SNP identification in GWAS, offering high accuracy and moderate computational requirements but limited to statistical inference without functional annotations. In contrast, RegVar provides robust tissue-specific regulatory predictions, leveraging multi-omics data, but requires significantly higher computational resources and high-quality tissue data. DeepWAS bridges GWAS and functional annotations effectively, offering insights into cell-type-specific mechanisms, but shares RegVar’s computational intensity and dependency on functional data. CADD, while not tissue-specific, provides broad applicability across coding and non-coding regions with moderate computational demands, making it a balanced tool for general prioritization tasks. For GWAS fine-mapping, FINEMAP is the most efficient. For tissue-specific analyses, RegVar is unparalleled but computationally expensive. DeepWAS is ideal for integrating GWAS with regulatory annotations, while CADD is best suited for comprehensive prioritization across multiple variant types. The choice of tool depends on research goals, dataset availability, and computational capacity. In generic risk analysis, integrating the outcomes of FINEMAP and CADD offers both precise causal variant identification and broad variant prioritization.

### Task 3: risk assessment and pathogenicity prediction

For early intervention through risk stratification, AI algorithms leverage various techniques that incorporate genetic risk variants in different loci to estimate an individual’s susceptibility to specific diseases [97]. Tools such as PRSice process both genotyped and imputed data, providing robust *p*-values, supporting multiple inheritance models, and enabling multi-trait analysis [98]. PolyPred combines PolyFun-pred and BOLT-LMM models to calculate PRS with enhanced cross-population accuracy. It integrates genome-wide, functionally informed fine-mapping and addresses population-specific LD differences. With an AUC > 0.92, PolyPred demonstrates high performance in risk prediction for diverse populations, particularly in non-European cohorts. However, it requires high-quality LD panels, imputed datasets, and large sample sizes, which may limit accessibility in resource-constrained settings. WEVar leverages ensemble-based machine learning to integrate outputs from multiple pathogenicity prediction tools such as CADD, SIFT, and PolyPhen-2, generating composite pathogenicity scores. Focused on SNP-level prioritization, WEVar is adaptable to diverse datasets and reduces bias from individual predictors. With an area under the receiver operating characteristic (AUROC) of 91% for non-coding functional prediction, it balances accuracy and computational efficiency. However, it is highly dependent on pre-computed scores and the quality of the integrated tools, making it less effective for novel variants not covered by existing databases.

At gene expression and cell-type analysis level, machine learning methods such as VariantSpark [73], GRAM [72], and MUSSEL [74] are used to uncover genetic associations, detect expression-modulating variants, and perform SNP selection for risk prediction in various diseases. These tools provide deeper insights into the functional consequences of genetic variants and their relationship to disease pathways. At the protein level, EVE is a generative model that predicts the pathogenicity of protein variants without labeled data by modeling sequence variation across organisms. GRAM focuses on predicting the expression-modulating effects of non-coding variants on associated genes. Using random forest and LASSO modeling, it integrates transcription factor binding scores and cell-type-specific gene expression profiles to compute expression-modulating effect scores. With a performance of 94% AUC in regulatory activity prediction, GRAM excels in gene-level non-coding variant analysis, offering high specificity for cell-type regulation. However, it struggles with 3′-terminal data and requires gene expression profiles and SELEX data for accurate predictions. It is computationally moderate, needing ∼16 GB RAM and training datasets from resources like GTEx and ENCODE.

In coding region, EVE uses a Bayesian Variational Autoencoder to predict the pathogenicity of protein-coding missense variants. It models evolutionary constraints from multiple sequence alignments (MSAs) and outputs pathogenicity scores and confidence classifications. With an AUC of ∼0.91 across 3219 disease-associated proteins, EVE excels in identifying disease-causing rare variants. However, it is limited to protein-coding regions, requiring high-quality MSAs, and does not handle splicing effects or variant combinations. Xrare integrates genetic evidence and phenotypic data using gradient boosting decision trees to prioritize variants, particularly in rare Mendelian diseases. Combining ACMG/AMP guidelines with phenotype similarity scores, it achieves an average accuracy of 95% for rare variant detection. It is highly sensitive to noisy phenotypic data and effective for both known and novel disease genes. However, Xrare is primarily focused on coding and proximal non-coding regions, with limited utility for deep non-coding variants.

In summary, GRAM and PolyPred specialize in polygenic and regulatory risk prediction, while WEVar assess variant pathogenicity. Xrare excels in rare disease variant prioritization, and EVE interprets protein-coding missense variants using evolutionary data. Performance varies, with PolyPred (AUC > 0.92) and CADD (98% accuracy) leading in risk prediction, while EVE and Xrare demonstrate high efficacy for specific variant types. Computational requirements range from moderate (e.g. GRAM, PolyPred) to high (e.g. RegVar, DeepWAS). Tools like Xrare and WEVar are computationally efficient, operating on standard setups. Strengths include GRAM’s cell-type-specific predictions and CADD’s broad variant scoring. Limitations stem from reliance on high-quality datasets (e.g. GRAM, RegVar) and restricted variant scopes (e.g. EVE, Xrare). Selection depends on study goals, datasets, and computational capacity, with CADD offering versatility for comprehensive analyses. The most practical approach for generic risk analysis integrates outcomes across tools, applies deep filtering to eliminate redundancies, and combines risk scores from multiple prediction methods across different omics levels for comprehensive and robust variant prioritization.

### Task 4: risk interpretation and network analysis

Graphical network methods provide robust capabilities for exploring genetic mechanisms, variant effects, and complex trait associations. MecCog employs graphical models to analyze disease mechanisms, integrating biomedical literature with real-world data to provide multi-omics insights into genes and pathways. With 90% biomarker identification accuracy, MecCog is computationally efficient, requiring only a standard laptop. However, its reliance on domain expertise for result interpretation can limit accessibility for less experienced users. It is best suited for non-coding, multi-omics analyses focused on understanding disease mechanisms. DeepHisCoM uses DL, specifically CNNs, to analyze pathways based on multi-omics data and GWAS integration. It offers precise pathway analysis with 94% precision, making it highly effective for identifying disease-associated pathways. However, the method demands extensive training data and high computational resources (>32 GB RAM and GPUs). DeepHisCoM is ideal for non-coding, SNP, and pathway-level analyses in research requiring robust pathway insights. SNPMap focuses on visualization and interpretation of SNP effects using statistical learning and automated pipelines. It provides intuitive visualization frameworks for SNP-level analysis with 85% accuracy, making it accessible for researchers with minimal computational infrastructure. However, SNPMap is limited to pre-existing annotations, restricting its application in novel datasets or exploratory analyses. Its low computational requirements make it suitable for both coding and non-coding variant visualization. GREEN-DB is a multiomics database that integrates multi-omics information to prioritize regulatory variants, leveraging machine learning for variant annotation and prioritization. With 90% annotation reliability, it provides deep insights into non-coding regions but requires comprehensive and high-quality datasets, limiting its scalability. Computationally moderate (∼16 GB RAM), GREEN-DB is a versatile tool for non-coding variant prioritization and multi-omics data integration. VariantSpark uses a random forest ensemble approach to identify complex trait associations, effectively handling SNP interactions and high-dimensional datasets. It achieves 85% prediction accuracy for complex traits but may lack interpretability due to the nature of ensemble methods. Requiring significant computational resources (>32 GB RAM), VariantSpark is best suited for studies involving multi-omics datasets and SNP-gene interactions across coding and non-coding regions.

Tools like MecCog and DeepHisCoM excel in disease mechanism interpretation and pathway analysis, while GREEN-DB and VariantSpark focus on multi-omics integration and complex trait analysis. SNPMap specializes in intuitive SNP effect visualization. DeepHisCoM leads in precision (94%), followed by MecCog (90% biomarker accuracy) and GREEN-DB (90% annotation reliability). Lightweight tools like MecCog and SNPMap operate on standard laptops, while DeepHisCoM and VariantSpark require high RAM and GPUs. Each tool has unique strengths: DeepHisCoM excels in pathway analysis, MecCog in disease mechanisms, GREEN-DB in multi-omics integration, VariantSpark in handling high-dimensional data, and SNPMap in user-friendly visualization. Limitations include reliance on domain expertise for MecCog, extensive training data for DeepHisCoM, and interpretability challenges for VariantSpark. The choice depends on the research focus, computational resources, and dataset availability, with DeepHisCoM suited for pathway analysis, MecCog for disease mechanisms, and GREEN-DB for non-coding variant prioritization. In generic risk analysis, interpreting the impact of genetic risk decisions requires multiple perspectives. Combining tools like MecCog for disease mechanism insights, DeepHisCoM for pathway-level analysis, and GREEN-DB for non-coding variant prioritization provides a robust approach. Integrating their outcomes with SNPMap for intuitive SNP visualization ensures comprehensive risk assessment across diverse biomedical systems, advancing precision medicine.

## Practical roadmap and workflow for implementing GRF analysis

**Figure 5.**
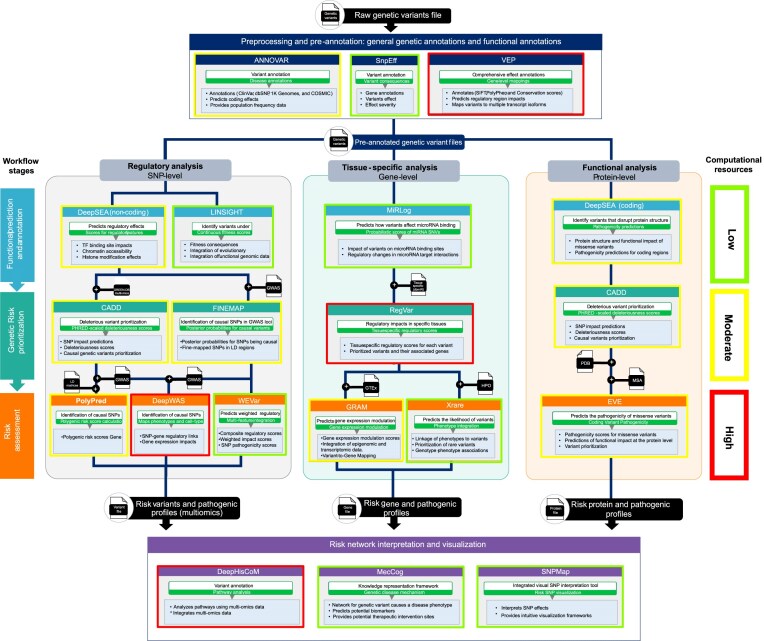
Practical workflow for GRF analysis. Comprehensive workflows for GRF analysis at three levels: regulatory analysis, gene-level functional prediction, and protein-level functional annotation. These workflows illustrate the integration of advanced AI tools, computational methods, and multi-omics datasets to optimize insights for precision medicine.

Based on our comparative analysis and ranking scores, we propose a practical roadmap to guide bioinformaticians in selecting and implementing the most effective 15 methods as shown in Fig. 5. We provide a guideline for implementing generic risk analysis at different levels of omics analysis (variant, gene, protein) by integrating the top-ranked (5-4) tools for each task. This approach facilitates enriched workflows for genetic risk analysis and precision medicine applications. The roadmap emphasizes robust data integration, accurate prediction models, and effective result interpretation to optimize patient outcomes and advance personalized healthcare. Fig. 5 illustrates the three main pipelines for analyzing GRFs: regulatory analysis (variant level), tissue-specific analysis (gene level), and functional and pathogenicity analysis (protein level). The workflows begin with raw variant files and progress through five stages, culminating in comprehensive network analysis. These stages include preprocessing and pre-annotation, functional annotation, genetic risk prioritization, risk assessment, and risk network interpretation and visualization. The computational cost of each method is highlighted: red indicates high computational demand, yellow represents moderate, and green signifies low resource requirements.

### Variant-level: regulatory analysis workflow

In the regulatory workflow, DeepSEA is used to predict regulatory effects such as transcription factor binding site impact and chromatin accessibility. LINSIGHT refines and filters variants based on fitness consequences in populations and evolutionary contexts, focusing on the analysis of genetic variants located in non-coding regions. GREEN-DB provides enriched multi-omics data, offering detailed information about controlled genes, tissues, and associated phenotypes to interpret the effects of variants across multi-omics levels. For risk prioritization, CADD and FINEMAP identify causative SNPs and deleterious variants, providing a ranking of their potential impacts. In risk assessment, PolyPred, DeepWAS, and WEVar are employed to score and compare the risk contributions of individual variants. In the final stage, the integrated variant profiles are analyzed using DeepHisCoM, MecCog, and SNPMap. These tools provide deeper insights into the importance of variants in disease mechanisms and the multilevel pathways that the variants influence, offering comprehensive interpretations of their role in genetic risk and disease progression.

### Gene-level: tissue-specific analysis

MiRLog is used to predict the effects of variants in microRNA regions and their impact on microRNA-target interactions. In the prioritization stage, RegVar assesses the regulatory impact of SNPs in specific tissues. For risk assessment, GRAM predicts gene expression modulation by integrating epigenomic and transcriptomic data, such as GTEx, while Xrare links phenotypes to rare variants by incorporating genotype-phenotype associations using Human Phenotype Ontology (HPO) terms to predict the likelihood of variant pathogenicity. Additionally, Xrare provides genotype-phenotype data visualization to facilitate interpretation. The integrated features from this stage feed into risk assessment tools to enable more comprehensive interpretation at the gene level. This workflow identifies critical gene-level associations and deepens our understanding of variant pathogenicity in multifactorial diseases.

### Protein level: functional and pathogenicity analysis

This workflow focuses on analyzing missense and other variants in coding regions to evaluate their impact on protein structure and function. DeepSEA is used to identify variants and predict structural disruptions caused by coding variants, as well as their pathogenicity. At the prioritization stage, CADD is employed to identify risk variants and their impact on protein function. For risk assessment, the generative model EVE utilizes protein structures derived from PDB to predict the pathogenic impact of variants on protein structure and function. In the final stage, integrating the outcomes of risk assessment with DeepHisCoM and MecCog to facilitate pathway analysis and the construction of protein networks that harbor harmful variants, offering valuable insights into disease mechanisms. This comprehensive approach aids in identifying potential biomarkers and suggests therapeutic targets, thereby advancing precision medicine strategies.

## Applications of optimized risk factors with AI in precision medicine

**Figure 6.**
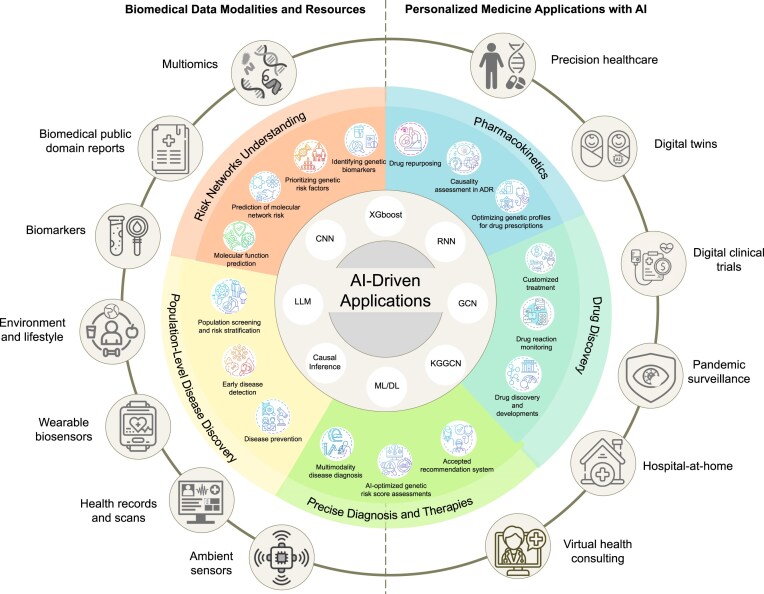
AI-driven applications of risk factors from difference modalities and resources in personalized medicine . This figure illustrates AI-driven applications of GRFs from various biomedical modalities in personalized medicine. It highlights the integration of multiple data sources such as multi-omics, health records, wearable biosensors, environmental and lifestyle data, virtual health consulting, digital twins, pandemic surveillance, hospital-at-home initiatives, and digital clinical trials to achieve precision healthcare. These AI technologies are applied to several medical applications including population screening, risk stratification, early disease detection, disease prevention, multimodal disease diagnosis, and AI-optimized genetic risk score assessments.

This section focuses on the practical applications and real-world impact of AI-optimized GRFs in precision medicine. By examining specific examples and case studies, we highlight how these advanced AI methods enhance precision medicine by offering treatments customized to individual patient needs. In Table 3, selected AI methods from Tables 1 and 2 have been experimentally validated through clinical or laboratory tests, underscoring key medical applications where GRFs critically influence disease prediction, prevention, and personalized treatment strategies. This section demonstrates the tangible benefits and potential of integrating AI-enhanced GRFs into clinical practice, showcasing the effectiveness of precision medicine. Additionally, the infographic in Fig. 6 presents AI-driven applications that harness biomedical resources to pinpoint risk factors, further enhancing precision medicine [3].

**Table 3.** Integration of AI methods in precision medicine

Medical tasks	Disease	Associated genetic factors	AI method	PMID
Patient stratification and screening	COVID-19, Asthma, Cardiovascular and Kidney Disease	ACE2, TMPRSS2, HLA-DQB1	Ensembl VEP, DSNetwork	38855114, 34880287
Advanced diagnostics	Complex Diseases, Cancer, Rheumatoid Arthritis	multi-omics, MAPK9, TNF, JAK inhibitors	Geneformer, PASNet	34579788
Risk assessment	Cardiovascular, Type 2 Diabetes, Cancer	PCSK9, APOB, LDLR, TCF7L2, PANK1	PolyPred, CADD, DeepSurv	37925478, 38256224, 38872215
Biomarker prediction	Coronary artery disease, Cancer	ANRIL, ARRDC3, SHBG	SNPnexus, RegBase, DeepSurv	36597873, 30412241, 38396861
Drug discovery and development	Cancer, Alzheimer’s Disease, Cystic Fibrosis, Familial Hypercholesterolemia	PIK3CA, BRCA1, BRCA2, APOE, CFTR, PCSK9, LDLR	CADD, SNP2TFBS, DeepVariant, PASNet	38822093, 33834176
Pharmacogenomics	Crohn disease, Hypertension	HLA, SEPHS1, GFRA2	SNPnexus, RegBase, DeepSurv	36597873, 30412241, 38396861
Discovering risk genes	Alzheimer’s Disease, Immune-mediated Diseases	PRL, PLCG2, ABI3, TREM2, LZTFL1, ATP2B1, NAV3, ITSN1, MARK2, SCAF1, HNRNPUL2	DeepSEA, GWAVA, DeepVariant	30820047, 28714976, 38816514, 35982159
Understanding disease mechanism	Alzheimer’s Disease, Immune-mediated Diseases	GRAMD1B, FUT2, ADAMTS4, HBEGF, WNT3, TSPAN14, DHODH, ABCB9, TNIP1	CADD, SNP2TFBS, MecCog	37841839, 36648426

### Integrative analysis of biomedical data for personalized medicine applications

The cornerstone of precision medicine lies in the integration of diverse biomedical data modalities [99]. For example, genomic data provide detailed profiles of patients’ genomes [100], electronic health records offer insights into past diagnoses and treatment responses [101], and wearable biosensor data track real-time health trends [102]. AI algorithms analyze these vast datasets to uncover patterns that may evade human interpretation, leading to significant advancements in diagnostics and personalized treatment plans [103]. By integrating biomedical resources, AI helps in identifying the root cause of illnesses with greater accuracy and tailors treatments to a patient’s genetic profile, enhancing effectiveness [104].

Furthermore, incorporating AI with digital twins (virtual representations of patients) [105] and pharmacokinetic data (describing drug behavior in the body) [106] allows for the simulation of treatment outcomes and the optimization of drug dosages tailored to individual genetic profiles, enhancing predictive modeling for personalized healthcare [107]. This approach extends to remote health consulting and hospital-at-home services, where ongoing patient monitoring and care are managed remotely [108]. These AI approaches turn diverse biomedical resources into actionable applications and personalized treatments, exemplifying the potential of personalized medicine.

### Precise disease detection and prevention

GRFs can identify individuals at higher risk for certain diseases before clinical indications occur, enabling personalized healthcare via population stratification [97]. Individuals at higher genetic risk benefit from more frequent and intensive screening, optimizing resource allocation and enhancing early disease detection [109]. In neurodegenerative disorders, GRFs facilitate early identification of Alzheimer’s disease by analyzing gene polymorphisms such as the APOE gene [110]. DL models predict Alzheimer’s progression based on APOE polymorphisms [111], and integrating AI with genetic and neuroimaging data assists in reading brain scans to identify Alzheimer’s genes [112]. AI techniques enhance cardiovascular health by identifying GRFs and integrating data from carotid ultrasound imaging and electronic health records [113]. For familial hypercholesterolemia, genetic markers such as PCSK9 and APOE guide early interventions [114]. AI also enhances hematopoietic disease diagnostics by automating cytomorphology, cytogenetics, and immunophenotyping, improving accuracy and reducing specialist workloads [115]. In type 2 diabetes, AI algorithms utilize GRFs such as TCF7L2 and lifestyle data for preemptive strategies [116]. AI integrates genetic and medical data to understand early T2D regulatory networks and uses GWAS for risk prediction [8, 9]. In kidney disease, an ML genetic programming algorithm improves early chronic kidney disease diagnosis by enhancing risk identification with a fitness function for imbalanced datasets [113, 117]. In oncology, genetic screening targets hereditary cancers, such as Hereditary Breast and Ovarian Cancer Syndrome (HBOC) and Lynch Syndrome using BRCA1 and BRCA2 mutations for earlier detection and better survival [118]. For hepatocellular carcinoma, early detection involves biomarkers such as circulating tumor DNA and microRNAs [119]. AI enhances cancer screening and supports lifestyle changes to mitigate risk, improving detection in breast, cervical, and prostate cancers [120].

### Precise diagnosis and improved patient outcomes

Several medical applications benefit from using GRFs with AI, providing advanced diagnostics and improving patient outcomes. In cancer research, AI identifies genomic biomarkers that respond to customized treatments, enhancing prognostic capabilities [70, 114, 121]. In drug target identification, GRFs pinpoint disease-associated genes or proteins, crucial for drug development [122]. For example, Cox-PASNet identifies clinically actionable genetic traits in glioblastoma multiforme and ovarian cancer, detecting key pathways and genes such as the PI3K cascade and MAPK9, which are crucial for tumor progression and potential therapy [69]. Moreover, AI and ML analyze gene data for personalized therapies with fewer side effects, applied in diseases such as IBD, colon cancer, AML, and breast cancer [123]. For cardiovascular diseases and type 2 diabetes, AI aids in patient care and predicts disease progression, improving outcomes and cost-effectiveness [112, 124]. For instance, the model DIABLO combines genetic and lifestyle data to predict diabetes onset and progression [125]. Similarly, in kidney diseases, the AI platform KidneyIntelX uses a ML algorithm to analyze data from electronic health records and blood tests to predict and manage disease progression, preventing organ failure and personalizing care [126]. Recent advancements in AI have transformed Alzheimer’s diagnostics by integrating multimodal data such as medical records, imaging, and novel biomarkers, offering a comprehensive approach to disease risk assessment [7, 27, 80]. For example, DeepAD analyzes MRI scans and genetic data in the APOE gene to improve early diagnosis and progression monitoring in Alzheimer’s disease [114]. These technologies also support the creation of clinical decision support systems that analyze patient data for personalized diagnostics and treatments [127].

### Targeted therapies and drug discovery

Utilizing GRFs alongside AI has significantly advanced treatment strategies across various diseases. For example, DeepCPI is used to identify compounds that act on drug targets for metabolic disorders and to screen drugs for hypertension [128]. In RA, genetic variations guide the development of targeted therapies such as TNF and JAK inhibitors [129]. Alzheimer’s disease benefits from AI by utilizing genetic variants in the APOE gene to guide therapies targeting amyloid-beta plaque accumulation [114]. In cystic fibrosis, treatments are tailored to specific Cystic Fibrosis Transmembrane Conductance Regulator (CFTR) gene mutations [130], and oncology leverages GRFs for targeted treatments in lung cancer, breast cancer, and chronic myeloid leukemia [5]. AI is crucial in neurogenetics, enhancing the understanding of complex disorders such as Alzheimer’s by annotating and prioritizing genetic factors, which informs therapeutic targets [111]. In pharmacogenomics, AI annotates genetic markers impacting drug metabolism to optimize medication dosage and reduce adverse reactions [131, 132]. Additionally, AI tailors anticoagulant therapy dosages based on GRFs, reducing risks associated with treatment [133]. In cancer research, AI innovations include noninvasive methods to detect Isocitrate Dehydrogenase (IDH) gene mutations and analyze lung tumor pathology images, enhancing precision in treatment and diagnostics [120]. AI’s role extends to identifying genetic causes in diseases such as inflammatory bowel disease and colon cancer, showcasing its versatility in drug target identification and therapy development [123].

### Pharmacokinetics and drug reactions

GRFs are integral to precision medicine, enabling targeted therapies and refined drug discovery [70]. For instance, HLA variants significantly increase the risk for diseases such as RA and lupus [134]. Early genetic testing for cystic fibrosis identifies individuals at risk, reducing potential organ failure [130]. Mayo Clinic’s use of AI and pharmacogenomics customizes RA medication by predicting responses to methotrexate using genomic and clinical data [135, 136]. Genetic profiles inform drug target identification and optimize dosages to enhance treatment response and minimize adverse reactions [137]. Variations in drug metabolism genes (ADME) illustrate the need for personalized dosages to mitigate side effects and enhance efficacy [138]. Moreover, genetic markers identify individuals at higher risk of adverse drug reactions, such as severe allergic responses to medications such as Carbamazepine [139]. The integration of AI with genetic data advances precision in drug metabolism prediction and therapy optimization [140, 141]. It helps predict individual drug metabolism variations and identifies markers for adverse reactions, improving patient safety and treatment efficacy [140].

### Understanding disease mechanisms and molecular risk networks

The integration of AI with GRFs significantly enhances our understanding of the molecular mechanisms underlying various diseases [7, 142, 143]. AI-driven analysis of genetic risk variants offers insights into predispositions across a broad spectrum of disorders, aiding in risk assessment and the development of personalized preventive strategies [7, 27]. AI-enhanced GRFs are crucial for understanding disease mechanisms, molecular networks, and for conducting functional genomics studies, which shape research priorities and deepen our knowledge of disease development [134, 136]. AI-driven tools, such as predictive networks, facilitate the analysis of gene interaction networks, enriching functional genomics research and aiding in the understanding of gene interactions and disease mechanisms [7, 144]. Combining GRFs with AI enhances predictive models for disease risk, facilitating early intervention and efficient healthcare allocation [145]. Tools such as STRING, DAVID, GeneMANIA, and Cytoscape enrich knowledge of molecular risk networks and disease mechanisms, advancing disease research and personalized treatments [7, 70]. The utilization of AI has helped researchers and clinicians uncover how genetic elements shape disease pathways, an essential aspect in crafting precise, targeted treatments. Both categories of genetic factors assume critical roles in precision medicine by providing essential insights into an individual’s genetic susceptibility to a range of health conditions [5, 134]. AI applications in genomics, such as pathway enrichment analysis and molecular risk network construction, reveal genes affected by risk variants and help in understanding disrupted cellular processes in disease development, thus contributing to precision medicine [70, 134, 136].

## Discussion and conclusion

The dawn of precision medicine marks a transformative era in healthcare, characterized by a shift from generalized treatment methodologies to highly individualized therapeutic strategies. Precision medicine relies heavily on vast amounts of biomedical data sourced from diverse resources, including wearable technology, medical data repositories with different modalities, multi-omics, epigenetics, and etiology data. This paradigm shift is enabled by the accurate representation and comprehensive analysis of biomedical big data to extract meaningful insights. AI plays a pivotal role in this process, capturing features and integrating GRFs using advanced algorithms such as generative AI, natural language processing, representation learning, causal models, reinforcement learning, and computer vision. This article provides a clear definition of GRFs and their role in enhancing precision medicine. It demonstrates how cutting-edge AI models can be utilized to integrate GRFs into downstream biomedical tasks effectively. A deep analysis and critical review of the top 20 validated methods are presented, evaluating their performance, applications, advantages, and limitations. A comprehensive comparison identifies the most effective tools for genetic risk assessment tasks, culminating in a proposed roadmap for utilizing the top 15 tools across omics levels. The roadmap emphasizes robust data integration, accurate prediction models, and effective result interpretation to advance personalized medicine. The article also explores the practical applications of AI in precision medicine, presenting insights through informative graphs that illustrate the impact of integrating AI, GRFs, and personalized healthcare. It highlights the ethical considerations in AI-driven GRF analysis and offers a comprehensive framework for incorporating advanced computational tools into clinical and research workflows. By addressing challenges in variant interpretation, multi-omics integration, and risk assessment, this study establishes a foundation for future innovations in precision medicine, emphasizing the need for interpretable, scalable AI models and equitable access to genomic technologies. Leveraging extensive biomedical public resources, AI facilitates novel advancements, paving the way for a healthcare paradigm that enhances outcomes and delivers personalized solutions tailored to individual genetic profiles.

## Data Availability

No new data were generated or analyzed in support of this research.

## References

[1] Akhmedov M, Martinelli A, Geiger R et al. Omics Playground: a comprehensive self-service platform for visualization, analytics and exploration of Big Omics Data. NAR Genom Bioinform. 2020; 2:lqz019 10.1093/nargab/lqz019. 33575569 PMC7671354

[2] Bachtiar M, Ooi BNS, Wang J et al. Towards precision medicine: interrogating the human genome to identify drug pathways associated with potentially functional, population-differentiated polymorphisms. Pharmacogenomics J. 2019; 19:516–27. 10.1038/s41397-019-0096-y. 31578463 PMC6867962

[3] Singh RS, Gupta BP Genes and genomes and unnecessary complexity in precision medicine. NPJ Genom Med. 2020; 5:21 10.1038/s41525-020-0128-1. 32377378 PMC7198588

[4] Kriger-Sharabi OA, Kopylov U Harnessing the power of precision medicine and novel biomarkers to treat Crohn’s Disease. J Clin Med. 2023; 12:2696 10.3390/jcm12072696. 37048779 PMC10094767

[5] Sathipati SY, Tsai MJ, Aimalla N et al. An evolutionary learning-based method for identifying a circulating miRNA signature for breast cancer diagnosis prediction. NAR Genom Bioinform. 2024; 6:lqae022 10.1093/nargab/lqae022. 38406797 PMC10894035

[6] Gu X, Jiang C, Zhao J et al. Identification of lipid metabolism-associated genes as prognostic biomarkers based on the immune microenvironment in hepatocellular carcinoma. Front Cell Dev Biol. 2022; 10:883059 10.3389/fcell.2022.883059. 36330335 PMC9622944

[7] Cuadrat RR, Kratzer A, Arnal HG et al. Cardiovascular disease biomarkers derived from circulating cell-free DNA methylation. NAR Genom Bioinform. 2023; 5:lqad061 10.1093/nargab/lqad061. 37388821 PMC10304763

[8] Choi S, Bae S, Park T Risk prediction using genome-wide association studies on type 2 diabetes. Genom Inform. 2016; 14:138 10.5808/GI.2016.14.4.138. PMC5287117 28154504

[9] Raimondi D, Simm J, Arany A et al. An interpretable low-complexity machine learning framework for robust exome-based in-silico diagnosis of Crohn’s disease patients. NAR Genom Bioinform. 2020; 2:lqaa011 10.1093/nargab/lqaa011. 33575557 PMC7671306

[10] Matheny ME, Whicher D, Israni ST Artificial intelligence in health care: a report from the National Academy of Medicine. Jama. 2020; 323:509–10. 10.1001/jama.2019.21579. 31845963

[11] Bianchi A, Zelli V, D’Angelo A et al. A method to comprehensively identify germline SNVs, INDELs and CNVs from whole exome sequencing data of BRCA1/2 negative breast cancer patients. NAR Genom Bioinform. 2024; 6:lqae033. 38633426 10.1093/nargab/lqae033 PMC11023157

[12] Gail HP, Scholz M, Pucci A Silicate condensation in Mira variables. Astron Astrophys. 2016; 591:A17.

[13] Erb I, Gloor GB, Quinn TP Compositional data analysis and related methods applied to genomics—a first special issue from NAR Genomics and Bioinformatics. NAR Genom Bioinform. 2020; 2:lqaa103. 33575646 10.1093/nargab/lqaa103 PMC7724639

[14] Xue Y, Lameijer EW, Ye K et al. Precision medicine: what challenges are we facing?. Genom Proteom Bioinform. 2016; 14:253–61. 10.1016/j.gpb.2016.10.001 PMC5093857 27744061

[15] Yang Y, Wang X, Xie K et al. kLDM: Inferring multiple metagenomic association networks based on the variation of environmental factors. Genom Proteom Bioinform. 2021; 19:834–47. 10.1016/j.gpb.2020.06.015 PMC9170748 33607296

[16] Chen F, Li C Inferring structural and dynamical properties of gene networks from data with deep learning. NAR Genom Bioinform. 2022; 4:lqac068. 36110897 10.1093/nargab/lqac068 PMC9469930

[17] Sahu M, Gupta R, Ambasta RK et al. Artificial intelligence and machine learning in precision medicine: A paradigm shift in big data analysis. Prog Mol Biol Trans Sci. 2022; 190:57–100. 10.1016/bs.pmbts.2022.03.002 36008002

[18] Wang D, Li J, Wang Y et al. A comparison on predicting functional impact of genomic variants. NAR Genom Bioinform. 2022; 4:lqab122. 35047814 10.1093/nargab/lqab122 PMC8759571

[19] Kent Jr JW Rare variants, common markers: synthetic association and beyond. Genet Epidemiol. 2011; 35:S80–4. 22128064 10.1002/gepi.20655 PMC3260649

[20] Chen W, Coombes BJ, Larson NB Recent advances and challenges of rare variant association analysis in the biobank sequencing era. Front Genet. 2022; 13:1014947. 36276986 10.3389/fgene.2022.1014947 PMC9582646

[21] Bodmer W, Bonilla C Common and rare variants in multifactorial susceptibility to common diseases. Nat Genet. 2008; 40:695–701. 10.1038/ng.f.136. 18509313 PMC2527050

[22] Williams RR Understanding genetic and environmental risk factors in susceptible persons. Western J Med. 1984; 141:799. PMC1011214 6596795

[23] Caiaffa CD, Fonteles CSR, Yunping L et al. Gene-environment interactions underlying the etiology of neural tube defects. Curr Top Dev Biol. 2023; 152:193. 36707212 10.1016/bs.ctdb.2022.10.007 PMC12619917

[24] Shoily SS, Ahsan T, Fatema K et al. Common genetic variants and pathways in diabetes and associated complications and vulnerability of populations with different ethnic origins. Sci Rep. 2021; 11:7504 10.1038/s41598-021-86801-2. 33820928 PMC8021559

[25] Zhao M, Havrilla JM, Fang L et al. Phen2Gene: rapid phenotype-driven gene prioritization for rare diseases. NAR Genom Bioinform. 2020; 2:lqaa032 10.1093/nargab/lqaa032. 32500119 PMC7252576

[26] Yuan J, Chen F, Fan D et al. EyeDiseases: an integrated resource for dedicating to genetic variants, gene expression and epigenetic factors of human eye diseases. NAR Genom Bioinform. 2021; 3:lqab050 10.1093/nargab/lqab050. 34085038 PMC8168129

[27] Abar L, Zuber V, Otto GW et al. Unravelling genetic architecture of circulatory amino acid levels, and their effect on risk of complex disorders. NAR Genom Bioinform. 2024; 6:lqae046 10.1093/nargab/lqae046. 38711861 PMC11071119

[28] MacArthur D, Manolio T, Dimmock D et al. Guidelines for investigating causality of sequence variants in human disease. Nature. 2014; 508:469–76. 10.1038/nature13127. 24759409 PMC4180223

[29] Ding K, de Andrade M, Manolio TA et al. Genetic variants that confer resistance to malaria are associated with red blood cell traits in African-Americans: an electronic medical record-based genome-wide association study. G3: Genes, Genomes, Genetics. 2013; 3:1061–8. 10.1534/g3.113.006452. 23696099 PMC3704235

[30] Zhang B, Gao X Deciphering DNA variant-associated aberrant splicing with the aid of RNA sequencing. Nat Genet. 2023; 55:732–3. 10.1038/s41588-023-01363-5. 37142847

[31] Huang C, Chen W, Wang X Studies on the fat mass and obesity-associated (FTO) gene and its impact on obesity-associated diseases. Genes Dis. 2023; 10:2351–65. 10.1016/j.gendis.2022.04.014. 37554175 PMC10404889

[32] Berger SL, Kouzarides T, Shiekhattar R et al. An operational definition of epigenetics. Genes Dev. 2009; 23:781–3. 10.1101/gad.1787609. 19339683 PMC3959995

[33] Maden SK, Thompson RF, Hansen KD et al. Human methylome variation across Infinium 450K data on the Gene Expression Omnibus. NAR Genom Bioinform. 2021; 3:lqab025 10.1093/nargab/lqab025. 33937763 PMC8061458

[34] Kapell S, Jakobsson ME Large-scale identification of protein histidine methylation in human cells. NAR Genom Bioinform. 2021; 3:lqab045 10.1093/nargab/lqab045. 34046594 PMC8140740

[35] Alsheikh AJ, Wollenhaupt S, King EA et al. The landscape of GWAS validation; systematic review identifying 309 validated non-coding variants across 130 human diseases. BMC Med Genom. 2022; 15:74 10.1186/s12920-022-01216-w. PMC8973751 35365203

[36] Wang Y, Jiang Y, Yao B et al. WEVar: a novel statistical learning framework for predicting noncoding regulatory variants. Brief Bioinform. 2021; 22:bbab189 10.1093/bib/bbab189. 34021560 PMC8574971

[37] Zhou J, Chen S, Wu Y et al. PPML-Omics: A privacy-preserving federated machine learning method protects patients’ privacy in omic data. Sci Adv. 2024; 10:eadh8601 10.1126/sciadv.adh8601. 38295178 PMC10830108

[38] Sheller MJ, Edwards B, Reina GA et al. Federated learning in medicine: facilitating multi-institutional collaborations without sharing patient data. Sci Rep. 2020; 10:12598 10.1038/s41598-020-69250-1. 32724046 PMC7387485

[39] Pattarabanjird T, Cress C, Nguyen A et al. A machine learning model utilizing a novel SNP shows enhanced prediction of coronary artery disease severity. Genes. 2020; 11:1446 10.3390/genes11121446. 33271747 PMC7760379

[40] Wu Y, Jing R, Dong Y et al. Functional annotation of sixty-five type-2 diabetes risk SNPs and its application in risk prediction. Sci Rep. 2017; 7:43709 10.1038/srep43709. 28262806 PMC5337961

[41] Schmal M, Girod C, Yaver D et al. A bioinformatic-assisted workflow for genome-wide identification of ncRNAs. NAR Genom Bioinform. 2022; 4:lqac059. 35979446 10.1093/nargab/lqac059 PMC9376865

[42] Peng J, Bao Z, Li J et al. DeepRisk: a deep learning approach for genome-wide assessment of common disease risk. Fundamental Research. 2024; 4:752–60. 39156563 10.1016/j.fmre.2024.02.015 PMC11330112

[43] Lan AY, Corces MR Deep learning approaches for noncoding variant prioritization in neurodegenerative diseases. Front Aging Neurosci. 2022; 14:1027224. 36466610 10.3389/fnagi.2022.1027224 PMC9716280

[44] Frazer J, Notin P, Dias M et al. Disease variant prediction with deep generative models of evolutionary data. Nature. 2021; 599:91–5. 10.1038/s41586-021-04043-8. 34707284

[45] Bartoszewicz JM, Seidel A, Renard BY Interpretable detection of novel human viruses from genome sequencing data. NAR Genom Bioinform. 2021; 3:lqab004 10.1093/nargab/lqab004. 33554119 PMC7849996

[46] Kundu K, Darden L, Moult J MecCog: a knowledge representation framework for genetic disease mechanism. Bioinformatics. 2021; 37:4180–6. 10.1093/bioinformatics/btab432. 34117883 PMC12161289

[47] Schubach M, Maass T, Nazaretyan L et al. CADD v1. 7: using protein language models, regulatory CNNs and other nucleotide-level scores to improve genome-wide variant predictions. Nucleic Acids Res. 2024; 52:D1143–54. 38183205 10.1093/nar/gkad989 PMC10767851

[48] Brandes N, Goldman G, Wang CH et al. Genome-wide prediction of disease variant effects with a deep protein language model. Nat Genet. 2023; 55:1512–22. 37563329 10.1038/s41588-023-01465-0 PMC10484790

[49] Piccialli F, Di Somma V, Giampaolo F et al. A survey on deep learning in medicine: why, how and when?. Inform Fusion. 2021; 66:111–37.

[50] Alrefaei AF, Hawsawi YM, Almaleki D et al. Genetic data sharing and artificial intelligence in the era of personalized medicine based on a cross-sectional analysis of the Saudi human genome program. Sci Rep. 2022; 12:1405. 35082362 10.1038/s41598-022-05296-7 PMC8791994

[51] Zhou J, Zhou L, Wang D et al. Personalized and privacy-preserving federated heterogeneous medical image analysis with pppml-hmi. Comput Biol Med. 2024; 169:107861 10.1016/j.compbiomed.2023.107861. 38141449

[52] Wilkinson MD, Dumontier M, Aalbersberg IJ et al. The FAIR Guiding Principles for scientific data management and stewardship. Scientific Data. 2016; 3:160018 10.1038/sdata.2016.18. 26978244 PMC4792175

[53] Klie A, Laub D, Talwar JV et al. Predictive analyses of regulatory sequences with EUGENe. Nat Comput Sci. 2023; 3:946–56. 10.1038/s43588-023-00544-w. 38177592 PMC10768637

[54] Zhao T, Wang F, Mott R et al. Using encrypted genotypes and phenotypes for collaborative genomic analyses to maintain data confidentiality. Genetics. 2024; 226:iyad210 10.1093/genetics/iyad210. 38085098 PMC11090459

[55] Long E, Wan P, Chen Q et al. From function to translation: decoding genetic susceptibility to human diseases via artificial intelligence. Cell Genom. 2023; 3:100320. 37388909 10.1016/j.xgen.2023.100320 PMC10300605

[56] O’Sullivan DE, Sutherland RL, Town S et al. Risk factors for early-onset colorectal cancer: a systematic review and meta-analysis. Clin Gastroenterol Hepatol. 2022; 20:1229–40. 10.1016/j.cgh.2021.01.037. 33524598

[57] Yoo JH, Jeong H, Chung TM Cutting-edge technologies for digital therapeutics: a review and architecture proposals for future directions. Appl Sci. 2023; 13:6929 10.3390/app13126929.

[58] Putzier M, Khakzad T, Dreischarf M et al. Implementation of cloud computing in the German healthcare system. NPJ Digit Med. 2024; 7:12 10.1038/s41746-024-01000-3. 38218892 PMC10787755

[59] Liang B, Gong H, Lu L et al. Risk stratification and pathway analysis based on graph neural network and interpretable algorithm. BMC Bioinform. 2022; 23:394 10.1186/s12859-022-04950-1. PMC9516820 36167504

[60] Arnold M, Raffler J, Pfeufer A et al. SNiPA: an interactive, genetic variant-centered annotation browser. Bioinformatics. 2015; 31:1334–6. 10.1093/bioinformatics/btu779. 25431330 PMC4393511

[61] Park C, Kim B, Park T DeepHisCoM: deep learning pathway analysis using hierarchical structural component models. Brief Bioinform. 2022; 23:bbac171 10.1093/bib/bbac171. 35598329

[62] Zhou J, Theesfeld CL, Yao K et al. Deep learning sequence-based ab initio prediction of variant effects on expression and disease risk. Nat Genet. 2018; 50:1171–9. 10.1038/s41588-018-0160-6. 30013180 PMC6094955

[63] Koscielny G, An P, Carvalho-Silva D et al. Open Targets: a platform for therapeutic target identification and validation. Nucleic Acids Res. 2017; 45:D985–94. 10.1093/nar/gkw1055. 27899665 PMC5210543

[64] Wang Y, Song C, Zhao J et al. SEdb 2.0: a comprehensive super-enhancer database of human and mouse. Nucleic Acids Res. 2023; 51:D280–90. 10.1093/nar/gkac968. 36318264 PMC9825585

[65] Akiyama M, Sakakibara Y Informative RNA base embedding for RNA structural alignment and clustering by deep representation learning. NAR Genom Bioinform. 2022; 4:lqac012 10.1093/nargab/lqac012. 35211670 PMC8862729

[66] Mieth B, Rozier A, Rodriguez JA et al. DeepCOMBI: explainable artificial intelligence for the analysis and discovery in genome-wide association studies. NAR Genom Bioinform. 2021; 3:lqab065 10.1093/nargab/lqab065. 34296082 PMC8291080

[67] Zhang J, Zhang L, Gongol B et al. spatialHeatmap: visualizing spatial bulk and single-cell assays in anatomical images. NAR Genom Bioinform. 2024; 6:lqae006 10.1093/nargab/lqae006. 38312938 PMC10836942

[68] Kuksa PP, Leung YY, Gangadharan P et al. FILER: a framework for harmonizing and querying large-scale functional genomics knowledge. NAR Genom Bioinform. 2022; 4:lqab123 10.1093/nargab/lqab123. 35047815 PMC8759563

[69] Hao J, Kim Y, Kim TK et al. PASNet: pathway-associated sparse deep neural network for prognosis prediction from high-throughput data. BMC bioinformatics. 2018; 19:510 10.1186/s12859-018-2500-z. 30558539 PMC6296065

[70] Campana PA, Prasse P, Lienhard M et al. Cancer drug sensitivity estimation using modular deep graph neural networks. NAR Genom Bioinform. 2024; 6:lqae043 10.1093/nargab/lqae043. 38680251 PMC11055499

[71] Arloth J, Eraslan G, Andlauer TF et al. DeepWAS: Multivariate genotype-phenotype associations by directly integrating regulatory information using deep learning. PLoS Comput Biol. 2020; 16:e1007616 10.1371/journal.pcbi.1007616. 32012148 PMC7043350

[72] Lou S, Cotter KA, Li T et al. GRAM: A generalized model to predict the molecular effect of a non-coding variant in a cell-type specific manner. PLoS Genet. 2019; 15:e1007860 10.1371/journal.pgen.1007860. 31469829 PMC6742416

[73] Bayat A, Szul P, O’Brien AR et al. VariantSpark: cloud-based machine learning for association study of complex phenotype and large-scale genomic data. GigaScience. 2020; 9:giaa077 10.1093/gigascience/giaa077. 32761098 PMC7407261

[74] Jin J, Zhan J, Zhang J et al. MUSSEL: enhanced Bayesian polygenic risk prediction leveraging information across multiple ancestry groups. Cell Genom. 2024; 4:100539 10.1016/j.xgen.2024.100539. 38604127 PMC11019365

[75] Long Y, Zhang B, Tian S et al. Accurate transcriptome-wide identification and quantification of alternative polyadenylation from RNA-seq data with APAIQ. Genome Res. 2023; 33:644–57. 10.1101/gr.277177.122. 37117035 PMC10234309

[76] Zhou J, Zhang B, Li H et al. Annotating TSSs in multiple cell types based on DNA sequence and RNA-seq data via DeeReCT-TSS. Genom Proteom Bioinform. 2022; 20:959–73. 10.1016/j.gpb.2022.11.010. PMC10025762 36528241

[77] Li Z, Li Y, Zhang B et al. DeeReCT-APA: prediction of alternative polyadenylation site usage through deep learning. Genom Proteom Bioinform. 2022; 20:483–95. 10.1016/j.gpb.2020.05.004. PMC9801043 33662629

[78] Xia Z, Li Y, Zhang B et al. DeeReCT-PolyA: a robust and generic deep learning method for PAS identification. Bioinformatics. 2019; 35:2371–9. 10.1093/bioinformatics/bty991. 30500881 PMC6612895

[79] Rogers MF, Shihab HA, Mort M et al. FATHMM-XF: accurate prediction of pathogenic point mutations via extended features. Bioinformatics. 2018; 34:511–3. 10.1093/bioinformatics/btx536. 28968714 PMC5860356

[80] Wang Z, Zhang Q, Lin JR et al. Deep post-GWAS analysis identifies potential risk genes and risk variants for Alzheimer’s disease, providing new insights into its disease mechanisms. Sci Rep. 2021; 11:20511 10.1038/s41598-021-99352-3. 34654853 PMC8519945

[81] Li X, Shi L, Wang Y et al. OncoBase: a platform for decoding regulatory somatic mutations in human cancers. Nucleic Acids Res. 2019; 47:D1044–55. 10.1093/nar/gky1139. 30445567 PMC6323961

[82] Zhang L, Shi J, Ouyang J et al. X-CNV: genome-wide prediction of the pathogenicity of copy number variations. Genome Med. 2021; 13:132 10.1186/s13073-021-00945-4. 34407882 PMC8375180

[83] Sharo AG, Hu Z, Sunyaev SR et al. StrVCTVRE: a supervised learning method to predict the pathogenicity of human genome structural variants. Am J Hum Genet. 2022; 109:195–209. 10.1016/j.ajhg.2021.12.007. 35032432 PMC8874149

[84] Gurbich TA, Ilinsky VV ClassifyCNV: a tool for clinical annotation of copy-number variants. Sci Rep. 2020; 10:20375 10.1038/s41598-020-76425-3. 33230148 PMC7683568

[85] Petrini A, Mesiti M, Schubach M et al. parSMURF, a high-performance computing tool for the genome-wide detection of pathogenic variants. GigaScience. 2020; 9:giaa052 10.1093/gigascience/giaa052. 32444882 PMC7244787

[86] Zhou H, Arapoglou T, Li X et al. FAVOR: functional annotation of variants online resource and annotator for variation across the human genome. Nucleic Acids Res. 2023; 51:D1300–11. 10.1093/nar/gkac966. 36350676 PMC9825437

[87] Fu Y, Liu Z, Lou S et al. FunSeq2: a framework for prioritizing noncoding regulatory variants in cancer. Genome Biol. 2014; 15:480 10.1186/s13059-014-0480-5. 25273974 PMC4203974

[88] Giacopuzzi E, Popitsch N, Taylor JC GREEN-DB: a framework for the annotation and prioritization of non-coding regulatory variants from whole-genome sequencing data. Nucleic Acids Res. 2022; 50:2522–35. 10.1093/nar/gkac130. 35234913 PMC8934622

[89] Khan A, Liu Q, Wang K iMEGES: integrated mental-disorder GEnome score by deep neural network for prioritizing the susceptibility genes for mental disorders in personal genomes. BMC Bioinform. 2018; 19:95–107. 10.1186/s12859-018-2469-7. PMC6309067 30591030

[90] Benner C, Spencer CC, Havulinna AS et al. FINEMAP: efficient variable selection using summary data from genome-wide association studies. Bioinformatics. 2016; 32:1493–501. 10.1093/bioinformatics/btw018. 26773131 PMC4866522

[91] Taylor KE, Ansel KM, Marson A et al. PICS2: next-generation fine mapping via probabilistic identification of causal SNPs. Bioinformatics. 2021; 37:3004–7. 10.1093/bioinformatics/btab122. 33624747 PMC8528038

[92] Wang J, Huang D, Zhou Y et al. CAUSALdb: a database for disease/trait causal variants identified using summary statistics of genome-wide association studies. Nucleic Acids Res. 2020; 48:D807–16. 31691819 10.1093/nar/gkz1026 PMC7145620

[93] Peng C, Dieck S, Schmid A et al. CADA: phenotype-driven gene prioritization based on a case-enriched knowledge graph. NAR Genom Bioinform. 2021; 3:lqab078 10.1093/nargab/lqab078. 34514393 PMC8415429

[94] de Langen P, Ballester B MUFFIN: a suite of tools for the analysis of functional sequencing data. NAR Genom Bioinform. 2024; 6:lqae051 10.1093/nargab/lqae051. 38745992 PMC11091926

[95] Sehgal D, Rathan ND, Özdemir F et al. Genomic wide association study and selective sweep analysis identify genes associated with improved yield under drought in Turkish winter wheat germplasm. Sci Rep. 2024; 14:8431 10.1038/s41598-024-57469-1. 38600135 PMC11006659

[96] Huang YF, Gulko B, Siepel A Fast, scalable prediction of deleterious noncoding variants from functional and population genomic data. Nat Genet. 2017; 49:618–24. 10.1038/ng.3810. 28288115 PMC5395419

[97] Torkamani A, Wineinger NE, Topol EJ The personal and clinical utility of polygenic risk scores. Nat Rev Genet. 2018; 19:581–90. 10.1038/s41576-018-0018-x. 29789686

[98] Choi SW, O’Reilly PF PRSice-2: Polygenic Risk Score software for biobank-scale data. Gigascience. 2019; 8:giz082 10.1093/gigascience/giz082. 31307061 PMC6629542

[99] Brechtmann F, Bechtler T, Londhe S et al. Evaluation of input data modality choices on functional gene embeddings. NAR Genom Bioinform. 2023; 5:lqad095 10.1093/nargab/lqad095. 37942285 PMC10629286

[100] Imbert S, Portejoie L, Pfister E et al. A multiplex PCR and DNA-sequencing workflow on serum for the diagnosis and species identification for invasive aspergillosis and mucormycosis. J Clin Microbiol. 2023; 61:e01409-22 10.1128/jcm.01409-22. 36533925 PMC9879116

[101] Shachak A, Hadas-Dayagi M, Ziv A et al. Primary care physicians’ use of an electronic medical record system: a cognitive task analysis. J Gen Intern Med. 2009; 24:341–8. 19130148 10.1007/s11606-008-0892-6 PMC2642564

[102] Saif N, Yan P, Niotis K et al. Feasibility of using a wearable biosensor device in patients at risk for Alzheimer’s disease dementia. J Prevent Alzheimer’s Dis. 2020; 7:104–11. 10.14283/jpad.2019.39 PMC8202529 32236399

[103] Ghaffar Nia N, Kaplanoglu E, Nasab A Evaluation of artificial intelligence techniques in disease diagnosis and prediction. Discov Artif Intell. 2023; 3:5. 10.1007/s44163-023-00049-5 PMC9885935 40478140

[104] El-Kamand S, Quinn JM, Sareen H et al. CRUX, a platform for visualising, exploring and analysing cancer genome cohort data. NAR Genom Bioinform. 2024; 6:lqae003. 38304083 10.1093/nargab/lqae003 PMC10833466

[105] Vallée A Envisioning the future of personalized medicine: role and realities of digital twins. J Med Internet Res. 2024; 26:e50204. 38739913 10.2196/50204 PMC11130780

[106] Benet LZ, Kroetz D, Sheiner L et al. Pharmacokinetics: the dynamics of drug absorption, distribution, metabolism, and elimination. Goodman and Gilman’s: The Pharmacological Basis of Therapeutics. 1996; New York, NY, USA McGraw-Hill e27.

[107] Zhu EY, Dupuy AJ Machine learning approach informs biology of cancer drug response. BMC Bioinform. 2022; 23:184. 10.1186/s12859-022-04720-z PMC9112473 35581546

[108] Tilhou AS, Jain A, DeLeire T Telehealth expansion, internet speed, and primary care access before and during COVID-19. JAMA Netw Open. 2024; 7:e2347686. 38180762 10.1001/jamanetworkopen.2023.47686 PMC10770767

[109] Vassy JL, Christensen KD, Schonman EF et al. The impact of whole-genome sequencing on the primary care and outcomes of healthy adult patients: a pilot randomized trial. Ann Intern Med. 2017; 167:159–69. 28654958 10.7326/M17-0188 PMC5856654

[110] Reitz C, Brayne C, Mayeux R Epidemiology of Alzheimer disease. Nat Rev Neurol. 2011; 7:137–52. 21304480 10.1038/nrneurol.2011.2 PMC3339565

[111] Mishra R, Li B The application of artificial intelligence in the genetic study of Alzheimer’s disease. Aging Dis. 2020; 11:1567. 33269107 10.14336/AD.2020.0312 PMC7673858

[112] Kozlov M AI that reads brain scans shows promise for finding Alzheimer’s genes. Nature. 2023; 10.1038/d41586-023-03482-9 37949989

[113] Yang NI, Yeh CH, Tsai TH et al. Artificial intelligence-assisted identification of genetic factors predisposing high-risk individuals to asymptomatic heart failure. Cells. 2021; 10:2430. 34572079 10.3390/cells10092430 PMC8470162

[114] Raulin AC, Doss SV, Trottier ZA et al. ApoE in Alzheimer’s disease: pathophysiology and therapeutic strategies. Mol Neurodegener. 2022; 17:72. 36348357 10.1186/s13024-022-00574-4 PMC9644639

[115] Walter W, Haferlach C, Nadarajah N et al. How artificial intelligence might disrupt diagnostics in hematology in the near future. Oncogene. 2021; 40:4271–80. 34103684 10.1038/s41388-021-01861-y PMC8225509

[116] Scott RA, Scott LJ, Mägi R et al. An expanded genome-wide association study of type 2 diabetes in Europeans. Diabetes. 2017; 66:2888–902. 28566273 10.2337/db16-1253 PMC5652602

[117] Kumar A, Sinha N, Bhardwaj A et al. Clinical risk assessment of chronic kidney disease patients using genetic programming. Comput Methods Biomech Biomed Engin. 2022; 25:887–95. 34726985 10.1080/10255842.2021.1985476

[118] Rebbeck TR, Lynch HT, Neuhausen SL et al. Prophylactic oophorectomy in carriers of BRCA1 or BRCA2 mutations. New Engl J Med. 2002; 346:1616–22. 12023993 10.1056/NEJMoa012158

[119] Monti R, Rautenstrauch P, Ghanbari M et al. Identifying interpretable gene-biomarker associations with functionally informed kernel-based tests in 190,000 exomes. Nat Commun. 2022; 13:5332. 36088354 10.1038/s41467-022-32864-2 PMC9464252

[120] Koh DM, Papanikolaou N, Bick U et al. Artificial intelligence and machine learning in cancer imaging. Commun Med. 2022; 2:133. 36310650 10.1038/s43856-022-00199-0 PMC9613681

[121] Kamel HFM, Al-Amodi HSAB Exploitation of gene expression and cancer biomarkers in paving the path to era of personalized medicine. Genom Proteom Bioinform. 2017; 15:220–35. 10.1016/j.gpb.2016.11.005 PMC5582794 28813639

[122] Floris M, Olla S, Schlessinger D et al. Genetic-driven druggable target identification and validation. Trends Genet. 2018; 34:558–70. 10.1016/j.tig.2018.04.004. 29803319 PMC6088790

[123] Vadapalli S, Abdelhalim H, Zeeshan S et al. Artificial intelligence and machine learning approaches using gene expression and variant data for personalized medicine. Brief Bioinform. 2022; 23:bbac191 10.1093/bib/bbac191. 35595537 PMC10233311

[124] Bai L, Zhang Y, Wang P et al. Improved diagnosis of rheumatoid arthritis using an artificial neural network. Sci Rep. 2022; 12:9810. 35697754 10.1038/s41598-022-13750-9 PMC9192742

[125] Rönn T, Perfilyev A, Oskolkov N et al. Predicting type 2 diabetes via machine learning integration of multiple omics from human pancreatic islets. Sci Rep. 2024; 14:14637. 38918439 10.1038/s41598-024-64846-3 PMC11199577

[126] Raihan MJ, Khan MAM, Kee SH et al. Detection of the chronic kidney disease using XGBoost classifier and explaining the influence of the attributes on the model using SHAP. Sci Rep. 2023; 13:6263. 37069256 10.1038/s41598-023-33525-0 PMC10110580

[127] Dias R, Torkamani A Artificial intelligence in clinical and genomic diagnostics. Genome Med. 2019; 11:70. 31744524 10.1186/s13073-019-0689-8 PMC6865045

[128] Wan F, Zhu Y, Hu H et al. DeepCPI: a deep learning-based framework for large-scale in silico drug screening. Genom Proteom Bioinform. 2019; 17:478–95. 10.1016/j.gpb.2019.04.003. PMC7056933 32035227

[129] McInnes IB, Schett G The pathogenesis of rheumatoid arthritis. New Engl J Med. 2011; 365:2205–19. 22150039 10.1056/NEJMra1004965

[130] Cutting GR Cystic fibrosis genetics: from molecular understanding to clinical application. Nat Genet. 2015; 16:45–56. 10.1038/nrg3849 PMC4364438 25404111

[131] Qureshi R, Irfan M, Gondal TM et al. AI in drug discovery and its clinical relevance. Heliyon. 2023; 9:e17575 10.1016/j.heliyon.2023.e17575. 37396052 PMC10302550

[132] Hamburg MA, Collins FS The path to personalized medicine. New Engl J Med. 2010; 363:301–4. 20551152 10.1056/NEJMp1006304

[133] Yeh CH, Chou YJ, Tsai TH et al. Artificial-intelligence-assisted discovery of genetic factors for precision medicine of antiplatelet therapy in diabetic peripheral artery disease. Biomedicines. 2022; 10:116. 35052795 10.3390/biomedicines10010116 PMC8773099

[134] Alsaedi SB, Mineta K, Gao X et al. Computational network analysis of host genetic risk variants of severe COVID-19. Human Genom. 2023; 17:17. 10.1186/s40246-023-00454-y PMC9977643 36859360

[135] Myasoedova E, Athreya AP, Crowson CS et al. Toward individualized prediction of response to methotrexate in early rheumatoid arthritis: a pharmacogenomics-driven machine learning approach. Arthr Care Res. 2022; 74:879–88. 10.1002/acr.24834 34902228

[136] Alsaedi S, Mineta K, Tamura N et al. Integrative multiomics network analysis of genetic risk factors to infer biomarkers and therapeutic targets for rheumatoid arthritis. Research Square5 January 2024, preprint: not peer reviewed 10.21203/rs.3.rs-3607429/v1. PMC12370121 40839671

[137] Wilke RA, Dolan ME Genetics and variable drug response. Jama. 2011; 306:306–7. 10.1001/jama.2011.998. 21771992 PMC3539154

[138] Relling MV, Evans WE Pharmacogenomics in the clinic. Nature. 2015; 526:343–50. 10.1038/nature15817. 26469045 PMC4711261

[139] Sukkarieh HH, Khokhar AA, Bustami RT et al. A highlight on carbamazepine-induced adverse drug reactions in Saudi Arabia: a retrospective medical records-based study. Naunyn-Schmiedeberg’s Arch Pharmacol. 2023; 396:3177–82. 37199768 10.1007/s00210-023-02525-2 PMC10567952

[140] Chen X, Guo Y, Chen X iGMDR: integrated Pharmacogenetic resource guide to cancer therapy and research. Genom Proteom Bioinform. 2020; 18:150–60. 10.1016/j.gpb.2019.11.011 PMC7646137 32916316

[141] Guo Z, Fu Y, Huang C et al. NOGEA: a network-oriented gene entropy approach for dissecting disease comorbidity and drug repositioning. Genom Proteom Bioinform. 2021; 19:549–64. 10.1016/j.gpb.2020.06.023. PMC9040018 33744433

[142] Cano-Gamez E, Trynka G From GWAS to function: using functional genomics to identify the mechanisms underlying complex diseases. Front Genet. 2020; 11:424. 32477401 10.3389/fgene.2020.00424 PMC7237642

[143] Claude E, Leclercq M, Thébault P et al. Optimizing hybrid ensemble feature selection strategies for transcriptomic biomarker discovery in complex diseases. NAR Genomics and Bioinformatics. 2024; 6:lqae079 10.1093/nargab/lqae079. 38993634 PMC11237901

[144] Wang H, Wang T, Zhao X et al. AI-Driver: an ensemble method for identifying driver mutations in personal cancer genomes. NAR Genom Bioinform. 2020; 2:lqaa084. 33575629 10.1093/nargab/lqaa084 PMC7671397

[145] Li X, Shi W, Zhang R et al. Integrate molecular phenome and polygenic interaction to detect the genetic risk of ischemic stroke. Front Cell Dev Biol. 2020; 8:453. 32671063 10.3389/fcell.2020.00453 PMC7326764

